# Supplementary material for: Demultiplexing and barcode-specific adaptive sampling for nanopore direct RNA sequencing
Source: Nat Commun. 2025 Apr 21;16:3742. doi: 10.1038/s41467-025-59102-9 (PMC12012114; doi:10.1038/s41467-025-59102-9)
Supplement: Supplementary file 1 — Supplementary Information [file 41467_2025_59102_MOESM1_ESM.pdf]

Demultiplexing and barcode-specific adaptive sampling for nanopore direct RNA sequencing

van der Toorn and Bohn *et al.*

Supplementary Table 1. Run-RNA-barcode to RTA assignments for RTA training/evaluation data with RNA002.

Data set	Sequencing run	RNA template	Replicate	RTA barcode	RNA barcode
1	1	*V. chol* glycine riboswitch	1	DPC_1<br>DPC_2<br>DPC_3<br>DPC_4	bc13<br>bc14<br>bc15<br>bc16
2	2	Tetrahymena ribozyme	2	DPC_1<br>DPC_2<br>DPC_3<br>DPC_4	bc04<br>bc05<br>bc17<br>BC18
3	3	*V. chol* glycine riboswitch	1	WDX_bc01<br>WDX_bc02<br>WDX_bc03<br>WDX_bc04<br>WDX_bc05<br>WDX_bc06<br>WDX_bc07<br>WDX_bc08<br>WDX_bc09<br>WDX_bc10<br>WDX_bc11<br>WDX_bc12	bc12<br>bc04<br>bc05<br>bc03<br>bc06<br>bc07<br>bc01<br>bc09<br>bc02<br>bc10<br>bc11<br>bc08
4	3	hc16	2	WDX_bc01<br>WDX_bc02<br>WDX_bc03<br>WDX_bc04<br>WDX_bc05<br>WDX_bc06<br>WDX_bc07<br>WDX_bc08<br>WDX_bc09<br>WDX_bc10<br>WDX_bc11<br>WDX_bc12	bc10<br>bc09<br>bc01<br>bc08<br>bc05<br>bc07<br>bc11<br>bc03<br>bc06<br>bc04<br>bc02<br>bc12
5	4	HCV IRES	3	WDX_bc01<br>WDX_bc02<br>WDX_bc03<br>WDX_bc04<br>WDX_bc05<br>WDX_bc06<br>WDX_bc07<br>WDX_bc08<br>WDX_bc09<br>WDX_bc10<br>WDX_bc11<br>WDX_bc12	bc05<br>bc06<br>bc07<br>bc01<br>bc11<br>bc03<br>bc04<br>bc10<br>bc02<br>bc09<br>bc08<br>bc12
6	4	bact RNase P (not poly-adenylated)	4	WDX_bc01<br>WDX_bc02<br>WDX_bc03<br>WDX_bc04<br>WDX_bc05<br>WDX_bc06<br>WDX_bc07<br>WDX_bc08<br>WDX_bc09<br>WDX_bc10<br>WDX_bc11<br>WDX_bc12	bc09<br>bc11<br>bc10<br>bc08<br>bc12<br>bc04<br>bc01<br>bc07<br>bc02<br>bc05<br>bc03<br>bc06

Supplementary Table 2. Generated experimental DPC-RTA data, coverage per barcode and replicate after mapping and adapter detection.

DPC-RTA	**Replicate 1** *V. chol* gly *Polyadenylated*	**Replicate 2** Tetrahymena Polyadenylated	**Total**
Barcode 1	601	1856	2457
Barcode 2	600	2482	3082
Barcode 3	191	3141	3332
Barcode 4	558	975	1533
Total	1950	8454	10404

Supplementary Table 3. Generated experimental WDX-RTA data for RNA002, coverage per barcode and replicate after mapping and adapter detection.

**WDX-RTA**	**Replicate 1** *V. chol* gly Polyadenylated	**Replicate 2** hc16 Polyadenylated	**Replicate 3** HCV IRES Polyadenylated	**Replicate 4** bact RNase P Not polyadenylated	**Total**
Barcode 01	548	2604	411	872	4435
Barcode 02	633	3493	712	1476	6314
Barcode 03	433	3125	868	894	5320
Barcode 04	652	3078	188	1182	5100
Barcode 05	111	2132	237	285	2765
Barcode 06	162	963	626	1480	3231
Barcode 07	888	4806	701	1207	7602
Barcode 08	3120	2962	703	1894	8679
Barcode 09	324	5839	427	1305	7895
Barcode 10	1006	2802	857	2073	6738
Barcode 11	588	2459	888	1649	5584
Barcode 12	570	5575	360	749	7254
Total	9035	39838	6978	15066	70917

Supplementary Table 4. DeePlexiCon RTA barcodes, sequences in 5' to 3' direction[1].

**Barcode ID**	**Barcode sequence**
Barcode 1	GGCTTCTTCTTGCTCTTAGG
Barcode 2	GTGATTCTCGTCTTTCTGCG
Barcode 3	GTACTTTTCTCTTTGCGCGG
Barcode 4	GGTCTTCGCTCGGTCTTATT

Supplementary Table 5. Comparative performance of model and barcode types at various confidence thresholds. Precision and recall as macro average across classes.

Model	Dataset	Reads classified as noise [%]	Confidence cutoff	Unclassfied reads [%]	Accuracy	Precision	Recall
DPC	1	0.000	0.000	0.000	0.873	0.860	0.853
DPC	1	0.000	0.300	6.634	0.905	0.896	0.886
DPC	1	0.000	0.500	11.020	0.925	0.919	0.907
DPC	1	0.000	0.800	19.751	0.956	0.952	0.944
DPC	2	0.000	0.000	0.000	0.833	0.785	0.846
DPC	2	0.000	0.300	9.164	0.878	0.831	0.891
DPC	2	0.000	0.500	15.916	0.906	0.859	0.918
DPC	2	0.000	0.800	26.206	0.954	0.916	0.963
WDX-DPC	1	0.000	0.000	0.000	0.932	0.915	0.938
WDX-DPC	1	0.000	0.300	5.179	0.951	0.939	0.954
WDX-DPC	1	0.000	0.500	9.487	0.965	0.957	0.967
WDX-DPC	1	0.000	0.800	18.462	0.982	0.977	0.983
WDX-DPC	2	0.000	0.000	0.000	0.937	0.929	0.919
WDX-DPC	2	0.000	0.300	4.093	0.957	0.950	0.944
WDX-DPC	2	0.000	0.500	7.275	0.969	0.964	0.958
WDX-DPC	2	0.000	0.800	14.443	0.985	0.980	0.979
WDX4	3-5	1.750	0.000	0.000	0.984	0.984	0.984
WDX4	3-5	1.750	0.300	1.375	0.987	0.987	0.987
WDX4	3-5	1.750	0.500	2.708	0.989	0.989	0.989
WDX4	3-5	1.750	0.800	6.083	0.992	0.992	0.992
WDX4	6	4.668	0.000	0.000	0.978	0.964	0.974
WDX4	6	4.668	0.300	2.541	0.984	0.978	0.980
WDX4	6	4.668	0.500	4.648	0.987	0.983	0.984
WDX4	6	4.668	0.800	9.977	0.990	0.990	0.988
WDX12	3-5	2.167	0.000	0.000	0.960	0.961	0.960
WDX12	3-5	2.167	0.300	3.750	0.976	0.976	0.976
WDX12	3-5	2.167	0.500	6.181	0.980	0.980	0.980
WDX12	3-5	2.167	0.800	13.444	0.986	0.986	0.986
WDX12	6	4.752	0.000	0.000	0.941	0.937	0.943
WDX12	6	4.752	0.300	6.000	0.965	0.963	0.965
WDX12	6	4.752	0.500	10.056	0.975	0.973	0.975
WDX12	6	4.752	0.800	19.010	0.984	0.983	0.983

Supplementary Table 6. Classification time per read for WarpDemuX models. Reported times reflect average time per read, computed from 10 runs of 4000 reads on 8 CPU cores (11th Gen Intel(R) Core(TM) i7–1165G7 (2.80GHz) with 32GB memory).

Model	Time [ms]
WDX4	1.61
WDX6	2.65
WDX8	4.05
WDX10	5.37
WDX12	6.68

Supplementary Table 7. Run time per read for WarpDemuX models. Reported times reflect average time per read, computed from 10 runs of 4000 reads on 8 CPU cores (11th Gen Intel(R) Core(TM) i7–1165G7 (2.80GHz) with 32GB memory). std: standard deviation.

Model	Mean runtime [ms]	std
WDX4	14.37	0.07
WDX6	14.62	0.12
WDX8	14.93	0.07
WDX10	15.26	0.09
WDX12	15.56	0.14

Supplementary Table 8. Optimized WarpDemuX barcodes, sequences in 5' to 3' direction.

Barcode ID	Barcode sequence
Barcode 1	TTTTTACTGCCAGTGACT
Barcode 2	AGGGGAGAGAGCCCCCCC
Barcode 3	CACGTCATTTTCCACGTC
Barcode 4	GGAGGCCAGGCGGACCGA
Barcode 5	ACGGACCTTTTGACTTAA
Barcode 6	TATTGCATACTGCGCCGC
Barcode 7	CCACGGAGGGAGGATTGG
Barcode 8	TTACCGGCAGTGACGGAC
Barcode 9	CGAGATTGCATCCCCCCC
Barcode 10	TACCACCTGCCGGCGGCC
Barcode 11	GCCCGCCGGGGGAGAAGC
Barcode 12	TTTTTTTTACCGGCAGTT

Supplementary Table 9. Best performing barcodes sets for various numbers of barcodes (RNA002), based on model selection benchmark.

Model	Number of samples	WarpDemuX barcodes
WDX4	4	4 5 6 8
WDX6	6	1 2 5 6 7 11
WDX8	8	1 3 5 6 7 9 11 12
WDX10	10	1 2 3 5 6 7 9 10 11 12
WDX12	12	1 2 3 4 5 6 7 8 9 10 11 12

Supplementary Table 10. Comparative performance of model and barcode types at stringent confidence thresholds 0.95. Precision and recall as macro average across classes. Stringent confidence cutoffs can be beneficial, for example in highly unbalanced samples in which the underrepresented sample is of interest.

Model	Dataset	Reads classified as noise [%]	Confidence cutoff	Unclassfied reads [%]	Accuracy	Precision	Recall
DPC	1	0.000	0.950	30.606	0.978	0.977	0.972
DPC	2	0.000	0.950	36.013	0.967	0.937	0.974
WDX-DPC	1	0.000	0.950	33.744	0.993	0.992	0.993
WDX-DPC	2	0.000	0.950	25.124	0.994	0.992	0.991
WDX4	3-5 (test set)	1.750	0.950	13.333	0.995	0.995	0.995
WDX4	6	4.668	0.950	19.335	0.993	0.993	0.991
WDX12	3-5 (test set)	2.167	0.950	31.194	0.991	0.990	0.991
WDX12	6	4.752	0.950	37.694	0.989	0.988	0.988

Supplementary Table 11. Comparative performance of model and barcode types at stringent confidence thresholds 0.99. Precision and recall as macro average across classes. Stringent confidence cutoffs can be beneficial, for example in highly unbalanced samples in which the underrepresented sample is of interest.

Model	Dataset	Reads classified as noise [%]	Confidence cutoff	Unclassfied reads [%]	Accuracy	Precision	Recall
DPC	1	0.000	0.990	43.270	0.989	0.987	0.986
DPC	2	0.000	0.990	48.392	0.981	0.952	0.985
WDX-DPC	1	0.000	0.990	50.564	0.994	0.994	0.991
WDX-DPC	2	0.000	0.990	40.040	0.997	0.998	0.995
WDX4	3-5 (test set)	1.750	0.990	21.792	0.995	0.994	0.995
WDX4	6	4.668	0.990	27.825	0.994	0.994	0.995
WDX12	3-5 (test set)	2.167	0.990	50.056	0.995	0.993	0.995
WDX12	6	4.752	0.990	54.500	0.989	0.986	0.988

Supplementary Table 12. Performance comparison across models in different operation modes.

Model	Dataset	Reads classified as noise [%]	mode	Confidence cutoff	Unclassfied reads [%]	Accuracy	Precision	Recall
WDX-DPC	1	0.0000	HR	0.1120	1.9000	0.9400	0.9250	0.9450
WDX-DPC	1	0.0000	HA	0.2250	4.1000	0.9460	0.9330	0.9500
WDX-DPC	2	0.0000	HR	0.1120	1.8000	0.9450	0.9380	0.9290
WDX-DPC	2	0.0000	HA	0.2250	3.0000	0.9510	0.9440	0.9370
WDX4	3-5	1.7500	HR	0.7700	5.5000	0.9924	0.9923	0.9924
WDX4	3-5	1.7500	HA	0.9400	11.0400	0.9943	0.9941	0.9943
WDX4	6	4.6700	HR	0.7700	9.1900	0.9899	0.9900	0.9864
WDX4	6	4.6700	HA	0.9400	16.9200	0.9926	0.9925	0.9917
WDX6	3-5	2.1900	HR	0.6600	4.6900	0.9949	0.9949	0.9949
WDX6	3-5	2.1900	HA	0.8900	10.5000	0.9968	0.9969	0.9968
WDX6	6	5.7300	HR	0.6600	8.6900	0.9921	0.9925	0.9899
WDX6	6	5.7300	HA	0.8900	16.2700	0.9952	0.9957	0.9940
WDX8	3-5	2.5600	HR	0.5600	4.7500	0.9912	0.9914	0.9911
WDX8	3-5	2.5600	HA	0.8200	9.4200	0.9948	0.9948	0.9947
WDX8	6	5.6000	HR	0.5600	7.9800	0.9851	0.9853	0.9834
WDX8	6	5.6000	HA	0.8200	14.6800	0.9900	0.9902	0.9897
WDX10	3-5	2.5200	HR	0.4835	5.2700	0.9870	0.9871	0.9869
WDX10	3-5	2.5200	HA	0.7370	10.0200	0.9924	0.9925	0.9922
WDX10	6	5.3100	HR	0.4835	8.5700	0.9827	0.9818	0.9813
WDX10	6	5.3100	HA	0.7370	14.6500	0.9890	0.9886	0.9877
WDX12	3-5	2.1700	HR	0.4315	5.5100	0.9788	0.9791	0.9785
WDX12	3-5	2.1700	HA	0.7200	10.9300	0.9853	0.9855	0.9850
WDX12	6	4.7500	HR	0.4315	8.8500	0.9717	0.9697	0.9717
WDX12	6	4.7500	HA	0.7200	15.7000	0.9811	0.9798	0.9805

Supplementary Table 13. Sequenced bases (sequence_length_template) of reads (mean_qscore_template>10) recovered with and without balancing for different balancing strategies (40 channels each). Values reported in 10'000 bases. No balancing indicates no adaptive-sampling, all reads are accepted. Adapter count balances based on relative counts of classified adapters. Read counts and Base counts strategies combine the relative barcode counts during adaptive sampling with information from the pod5 files created during sequencing to correct relative counts for adapter-only reads (discarding adapter-only reads). Base counts takes into account the estimated bases sequenced per barcode based on the read lengths in the pod5 file. WDX label 'unclassified' refers to signal not confidently classified as one of the training adapters (confidence score threshold for filtering can be set at run time, here:0.4). WDX label 'outlier' refers to reads where the signal is considered an outlier with respect to the training signal distribution (noise-class prediction).

WDX label	No balancing	Adapter count	Read count	Base count
bc01	72.55	30.17	44.37	52.49
bc02	48.53	32.45	49.55	50.59
bc03	140.17	41.91	53.04	50.14
bc04	501.98	53.91	58.50	47.31
bc05	360.85	47.28	53.67	52.92
bc06	6.68	12.74	12.78	15.71
bc07	72.25	38.18	47.87	49.38
bc08	46.59	41.88	46.72	52.05
bc09	154.61	44.42	55.13	54.84
bc10	221.41	54.27	65.01	53.31
bc11	186.37	48.86	55.42	47.99
bc12	4.85	7.83	7.34	9.98
unclassified	79.76	118.84	125.51	112.62
outlier	46.70	44.83	39.66	84.58

Supplementary Table 14. Run-RNA-barcode to RTA assignments for RTA training/evaluation data with RNA004.

Data set	Sequencing run	RNA template	Replicate	RTA barcode	RNA barcode
7	5	HCV IRES	1	WDX_bc01	bc01
				WDX_bc02	bc02
				WDX_bc03	bc03
				WDX_bc04	bc04
				WDX_bc05	bc05
				WDX_bc06	bc06
				WDX_bc07	bc07
				WDX_bc08	bc08
				WDX_bc09	bc09
				WDX_bc10	bc10
				WDX_bc11	bc11
				WDX_bc12	bc12
8	5	*V. chol* glycine riboswitch	2	WDX_bc01	bc12
				WDX_bc02	bc11
				WDX_bc03	bc10
				WDX_bc04	bc09
				WDX_bc05	bc08
				WDX_bc06	bc07
				WDX_bc07	bc06
				WDX_bc08	bc05
				WDX_bc09	bc04
				WDX_bc10	bc03
				WDX_bc11	bc02
				WDX_bc12	bc01
9	5	bact. RNase P	3	WDX_bc01	bc09
				WDX_bc02	bc10
				WDX_bc03	bc11
				WDX_bc04	bc12
				WDX_bc05	bc01
				WDX_bc06	bc02
				WDX_bc07	bc03
				WDX_bc08	bc04
				WDX_bc09	bc05
				WDX_bc10	bc06
				WDX_bc11	bc07
				WDX_bc12	bc08
10	5	hc16	4	WDX_bc01	bc05
				WDX_bc02	bc06
				WDX_bc03	bc07
				WDX_bc04	bc08
				WDX_bc05	bc09
				WDX_bc06	bc10
				WDX_bc07	bc11
				WDX_bc08	bc12
				WDX_bc09	bc01
				WDX_bc10	bc02
				WDX_bc11	bc03
				WDX_bc12	bc04
11	5	curlcake1	5	WDX_bc01	bc02
				WDX_bc02	bc03
				WDX_bc03	bc04
		curlcake2		WDX_bc04	bc05
				WDX_bc05	bc06
				WDX_bc06	bc07
		curlcake3		WDX_bc07	bc08
				WDX_bc08	bc09
				WDX_bc09	bc10
		curlcake4		WDX_bc10	bc11
				WDX_bc11	bc12
				WDX_bc12	bc01

Supplementary Table 15. Generated experimental WDX-RTA data for RNA004, coverage per barcode and replicate after mapping and adapter detection.

**WDX-RTA**	**Replicate 1** HCV IRES	**Replicate 2** *V. chol* gly	**Replicate 3** bact RNase P	**Replicate 4** hc16	**Replicate 5** curlcake1	curlcake2	curlcake3	curlcake4	**Total**
Barcode1	17583	14462	22919	18504	78	61	77	72	73756
Barcode2	13215	11574	20402	13805	83	73	65	74	59291
Barcode3	19429	13722	26849	21784	152	109	104	124	82273
Barcode4	17172	14307	37345	20534	74	65	63	67	89627
Barcode5	19165	17904	28624	17538	108	73	70	63	83545
Barcode6	19058	13692	21712	17794	80	68	42	53	72499
Barcode7	19151	15474	18877	10366	168	127	101	126	64390
Barcode8	32619	10394	19809	16254	139	116	118	122	79571
Barcode9	22059	10807	14998	13427	126	99	111	92	61719
Barcode10	17935	12974	18713	19945	175	141	160	148	70191
Barcode11	17106	13661	19312	24613	102	80	66	85	75025
Barcode12	27768	13928	21410	22037	109	91	91	89	85523
Total	242260	162899	270970	216601	1394	1103	1068	1115	897410

Supplementary Table 16. WarpDemuX barcodes per trained RNA004 model.

Model	Number of samples	WarpDemuX barcodes
WDX4	4	3 4 5 7
WDX6	6	4 5 6 7 11 12
WDX8	8	3 4 5 6 7 9 11 12
WDX10	10	3 4 5 6 7 8 9 10 11 12
WDX12	12	1 2 3 4 5 6 7 8 9 10 11 12

Supplementary Table 17. WDX4 RNA004 per barcode performance.

WDX-RTA	Dataset	Targeted accuracy							Target accuracy						
		0.95	0.96	0.97	0.98	0.99	0.995	0.999	0.95	0.96	0.97	0.98	0.99	0.995	0.999
		Observed accuracy							Unclassified reads (%)						
Barcode 03	Test set 1	0.982	0.982	0.982	0.982	0.989	0.993	0.996	0.1	0.1	0.1	0.1	1.4	2.8	4.7
	Test set 2	0.986	0.986	0.986	0.986	0.991	0.994	0.997	0.0	0.0	0.0	0.0	1.0	2.5	4.3
Barcode 04	Test set 1	0.979	0.979	0.979	0.979	0.988	0.996	0.999	0.1	0.1	0.1	0.1	1.9	4.8	37.5
	Test set 2	0.980	0.980	0.980	0.980	0.989	0.997	1.000	0.2	0.2	0.2	0.2	2.2	5.0	38.6
Barcode 05	Test set 1	0.973	0.973	0.973	0.973	0.982	0.992	0.998	0.0	0.0	0.0	0.2	2.5	6.4	20.2
	Test set 2	0.969	0.969	0.969	0.970	0.981	0.991	0.996	0.1	0.1	0.1	0.3	2.4	6.0	20.1
Barcode 07	Test set 1	0.974	0.974	0.974	0.982	0.988	0.993	0.996	0.3	0.3	0.3	2.4	4.6	8.7	56.4
	Test set 2	0.974	0.974	0.974	0.981	0.987	0.993	0.999	0.2	0.2	0.2	2.4	4.7	8.6	56.9
Average	Test set 1	0.977	0. 977	0.977	0.979	0.987	0.993	0.997	0.1	0.1	0.1	0.7	2.6	5.7	29.7
	Test set 2	0.977	0. 977	0.977	0.979	0.987	0.994	0.998	0.1	0.1	0.1	0.7	2.6	5.5	30.0

Supplementary Table 18. WDX10 RNA004 per barcode performance.

WDX-RTA	Dataset	Target accuracy							Target accuracy						
		0.95	0.96	0.97	0.98	0.99	0.995	0.999	0.95	0.96	0.97	0.98	0.99	0.995	0.999
		Observed accuracy							Unclassified reads (%)						
Barcode 03	Test set 2	0.955	0.964	0.975	0.986	0.996	0.998	0.998	0.8	2.0	4.2	7.8	19.9	41.8	58.7
	Test set 1	0.951	0.958	0.968	0.980	0.989	0.991	0.991	0.9	2.2	4.2	7.5	18.7	41.3	58.7
Barcode 04	Test set 2	0.958	0.958	0.960	0.974	0.990	0.995	0.997	0.1	0.1	0.5	3.0	9.2	20.8	62.8
	Test set 1	0.960	0.960	0.963	0.975	0.991	0.995	0.995	0.1	0.1	0.6	2.8	8.7	19.4	62.1
Barcode 05	Test set 2	0.957	0.957	0.964	0.975	0.988	0.994	0.997	0.0	0.0	1.2	3.1	6.8	12.8	37.2
	Test set 1	0.960	0.960	0.968	0.979	0.990	0.996	0.998	0.0	0.0	1.5	3.4	7.0	13.1	37.3
Barcode 06	Test set 1	0.953	0.953	0.964	0.978	0.986	0.991	0.997	0.2	0.2	2.1	5.6	9.1	14.3	51.2
	Test set 2	0.950	0.950	0.963	0.977	0.986	0.993	0.997	0.1	0.1	2.3	6.0	9.4	15.3	53.2
Barcode 07	Test set 2	0.960	0.960	0.968	0.977	0.985	0.991	0.995	0.0	0.0	1.7	4.0	6.8	12.6	92.7
	Test set 1	0.962	0.962	0.971	0.978	0.985	0.990	0.990	0.0	0.0	1.7	3.6	6.8	12.3	93.1
Barcode 08	Test set 1	0.952	0.957	0.963	0.972	0.987	0.990	0.990	1.2	1.9	3.1	5.6	16.9	38.0	62.0
	Test set 2	0.949	0.954	0.961	0.972	0.991	0.995	0.995	1.1	1.9	3.3	6.1	18.0	39.4	63.5
Barcode 09	Test set 1	0.950	0.957	0.966	0.973	0.985	0.991	0.995	0.0	1.2	2.7	4.4	9.1	15.3	59.3
	Test set 2	0.949	0.957	0.965	0.976	0.989	0.996	0.998	0.0	1.4	2.9	5.0	9.9	16.1	60.8
Barcode 10	Test Set 2	0.952	0.962	0.978	0.988	0.993	0.995	0.997	2.7	4.8	9.2	14.2	21.5	27.5	75.6
	Test Set 1	0.955	0.963	0.978	0.984	0.991	0.993	0.990	2.8	4.7	9.3	13.6	20.8	26.3	74.9
Barcode 11	Test set 1	0.954	0.965	0.976	0.982	0.988	0.993	0.995	1.5	3.9	7.7	11.9	18.6	32.9	51.7
	Test set 2	0.956	0.969	0.979	0.986	0.991	0.994	0.996	1.3	4.0	7.7	12.1	18.4	32.5	50.5
Barcode 12	Test set 1	0.952	0.956	0.965	0.974	0.985	0.992	0.996	0.0	0.8	2.6	4.7	9.5	15.0	32.5
	Test set 2	0.952	0.957	0.966	0.976	0.987	0.994	0.997	0.0	0.9	2.8	5.0	9.7	15.2	33.3
Average	Test set 1	0.955	0.959	0.968	0.978	0.988	0.992	0.994	0.7	1.5	3.6	6.3	12.5	22.8	58.3
	Test set 2	0.954	0.959	0.968	0.979	0.990	0.994	0.997	0.6	1.5	3.6	6.6	13.0	23.4	58.8

Supplementary Table 19. Latency of basecalling for RNA adaptive sampling (AS) at 0.1s data chunks, with 500–4000 samples per read at time of basecalling across different system configurations. OS and N indicate respectively the Operating System used and the number of basecalling executions measured. Std: standard deviation.

	Model AS	Model live	OS	Mean (ms)	std	N
Case 1	fast	-	Linux	427.94	5.12	1322
			Windows	524.54	14.45	1133
Case 2	hac	—	Windows	807.67	33.05	742
Case 3	hac	hac	Windows	1064.39	247.34	574

Supplementary Table 20. Expected maximal yield per sample, without sample multiplexing, assuming flowcells can be washed (reused) 5 times.

Number of samples	Number of flowcells	Number of washes	Expected yield per sample (Gb) MinION (10Gb)	PromethION (50Gb)
2	1	1	25.0	145.0
4	1	3	12.5	72.5
6	2	4	16.7	96.7
8	2	6	12.5	72.5
10	2	8	10.0	58.0
12	3	9	12.5	72.5

Supplementary Table 21. Expected maximal yield per sample, with sample multiplexing, assuming yield of demultiplexing to be 85%.

Number of samples	Number of flowcells	Number of washes	Expected yield per sample (Gb) MinION (10Gb)	PromethION (50Gb)
2	1	0	21.3	123.3
4	1	0	10.6	61.6
6	1	0	7.1	41.1
8	1	0	5.3	30.8
10	1	0	4.3	24.7
12	1	0	3.5	20.5

Supplementary Table 22. Absolute savings of sample multiplexing compared to sequencing without multiplexing.

Number of samples	Cost per sample	Cost per experiment	Cost per Gb	Time (hr:min)
2	79 €	159 €	-1.64 €	2:15
4	112 €	450 €	4.17 €	5:15
6	252 €	1,510 €	0.59 €	4:00
8	225 €	1,801 €	3.47 €	6:00
10	209 €	2,092 €	6.36 €	8:00
12	263 €	3,152 €	3.17 €	7:25

Supplementary Table 23. Relative savings of sample multiplexing compared to sequencing without multiplexing.

Number of multiplexed samples	Cost per sample	Cost per experiment	Cost per Gb	Time
2	14%	14%	-7%	43%
4	32%	32%	15%	64%
6	61%	61%	2%	57%
8	65%	65%	13%	67%
10	68%	68%	21%	73%
12	76%	76%	12%	71%

Supplementary Table 24. Custom RTA design scheme (5’ to 3’ orientation for both oligos).

Forward adapter	/5Phos/GG–[WDX barcode]–GGTAGTAGGTTC
Reverse adapter	GAGGCGAGCGGTCAATTTT–[revcomp–WDX barcode]–CCTTTTTTTTTT

Supplementary Table 25. RNA barcode design and reverse PCR primers to generate *in vitro* transcription templates. The constant M13r sequence is in lowercase.

RNA barcode ID	Barcode unique sequence	Reverse primer for PCR
RNA_bc01	GATAGTGCgagtaGCACTATC	TTTTTTTTTTGATAGTGCTACTCGCACTATCcaggaaacagctatgaccatg
RNA_bc02	GCAATACTgagtaAGTATTGC	TTTTTTTTTTGCAATACTTACTCAGTATTGCcaggaaacagctatgaccatg
RNA_bc03	GATTATCAgagtaTGATAATC	TTTTTTTTTTGATTATCATACTCTGATAATCcaggaaacagctatgaccatg
RNA_bc04	AGGTCAGGgagtaCCTGACCT	TTTTTTTTTTAGGTCAGGTACTCCCTGACCTcaggaaacagctatgaccatg
RNA_bc05	GAGGCTCCgagtaGGAGCCTC	TTTTTTTTTTGAGGCTCCTACTCGGAGCCTCcaggaaacagctatgaccatg
RNA_bc06	GCAACCAGgagtaCTGGTTGC	TTTTTTTTTTGCAACCAGTACTCCTGGTTGCcaggaaacagctatgaccatg
RNA_bc07	GACTCCATgagtaATGGAGTC	TTTTTTTTTTGACTCCATTACTCATGGAGTCcaggaaacagctatgaccatg
RNA_bc08	GTGTAATCgagtaGATTACAC	TTTTTTTTTTGTGTAATCTACTCGATTACACcaggaaacagctatgaccatg
RNA_bc09	ATGACGAAgagtaTTCGTCAT	TTTTTTTTTTATGACGAATACTCTTCGTCATcaggaaacagctatgaccatg
RNA_bc10	CATGTTGAgagtaTCAACATG	TTTTTTTTTTCATGTTGATACTCTCAACATGcaggaaacagctatgaccatg
RNA_bc11	CGAACTTCgagtaGAAGTTCG	TTTTTTTTTTCGAACTTCTACTCGAAGTTCGcaggaaacagctatgaccatg
RNA_bc12	TATCTGTGgagtaCACAGATA	TTTTTTTTTTTTGAGTACTACTCGTACTCAAcaggaaacagctatgaccatg
RNA_bc13	GACGGACCgagtaGGTCCGTC	TTTTTTTTTTGACGGACCTACTCGGTCCGTCcaggaaacagctatgaccatg
RNA_bc14	GAGGTCTGgagtaCAGACCTC	TTTTTTTTTTGAGGTCTGTACTCCAGACCTCcaggaaacagctatgaccatg
RNA_bc15	GCTCAATCgagtaGATTGAGC	TTTTTTTTTTGCTCAATCTACTCGATTGAGCcaggaaacagctatgaccatg
RNA_bc16	GACGATACgagtaGTATCGTC	TTTTTTTTTTGACGATACTACTCGTATCGTCcaggaaacagctatgaccatg
RNA_bc17	GACCGCCTgagtaAGGCGGTC	TTTTTTTTTTGACCGCCTTACTCAGGCGGTCcaggaaacagctatgaccatg
RNA_bc18	GAGTCCTTgagtaAAGGACTC	TTTTTTTTTTGAGTCCTTTACTCAAGGACTCcaggaaacagctatgaccatg

Supplementary Table 26. Forward PCR primers to generate *in vitro* transcription templates.

Template	Forward primer sequence
HCV IRES	AAAGAAGACTTGGGGTAATACGACTCACTATAGGCCAGCCCCCGATTG
V. chol glycine riboswitch	TTCTAATACGACTCACTATAGGTCCGTTGAAGACTGCAGGAGAGTGG
bact Rnase P	TTCTAATACGACTCACTATAGGAGAGGAGCAGGC
hc16	TTCTAATACGACTCACTATAGGTAGACTCGCAGGAAGTCTACCGAGTAAGAGAAAGAGGA
Tetrahymena ribozyme	TTCTAATACGACTCACTATAGGAGGGAAAAGTTATCAGGCATGCACCTGGT

Supplementary Table 27. WarpDemuX RTA adapter to sample assignment of SARS-CoV-2 sequencing run.

Sequencing run	WDX barcode	Sample	Location
5	WDX_bc01	Vero-TMPRSS2 mock	cell
	WDX_bc02	Vero-TMPRSS2 inf. with SARS-CoV-2 8 h p.i.	cell
	WDX_bc03	Vero-TMPRSS2 inf. with SARS-CoV-2 24 h p.i.	cell
	WDX_bc04	Vero-TMPRSS2 inf. with SARS-CoV-2 48 h p.i.m	cell
	WDX_bc05	Vero-TMPRSS2 inf. with SARS-CoV-2 72 h p.i.	cell
	WDX_bc06	Vero-TMPRSS2 inf. with SARS-CoV-2 72 h p.i.	supernatant
	WDX_bc07	Vero-TMPRSS2 mock	cell
	WDX_bc08	Vero-TMPRSS2 inf. with SARS-CoV-2-GFP 8 h p.i.	cell
	WDX_bc09	Vero-TMPRSS2 inf. with SARS-CoV-2-GFP 24 h p.i.	cell
	WDX_bc10	Vero-TMPRSS2 inf. with SARS-CoV-2-GFP 48 h p.i.	cell
	WDX_bc11	Vero-TMPRSS2 inf. with SARS-CoV-2-GFP 72 h p.i.	cell
	WDX_bc12	Vero-TMPRSS2 inf. with SARS-CoV-2-GFP 72 h p.i.	supernatant

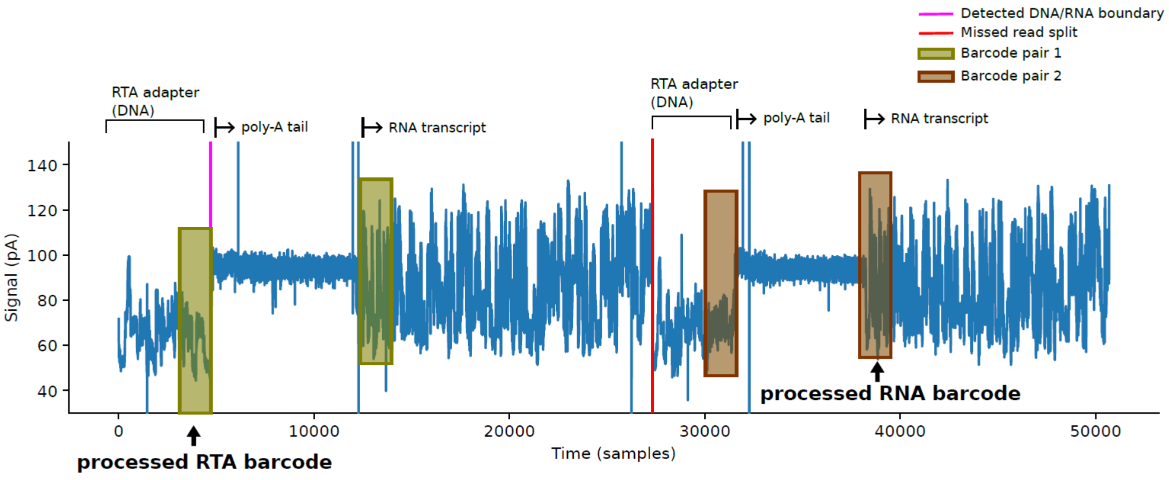

(a) Case 1

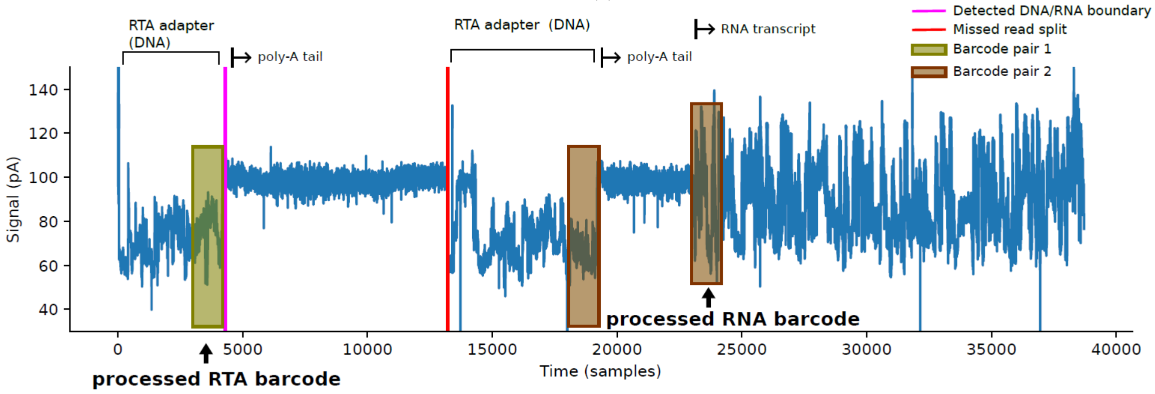

(b) Case 2

Supplementary Figure 1. Examples of mislabeled signal instances due to missed read splits. (a) Concatenation of two complete reads, each consisting of an adapter, polyA and RNA signal. (b) Concatenation of an 'adapter-only' read and a complete read, wherein the first consists of an adapter and subsequent polyA tail signal, but misses the RNA signal.

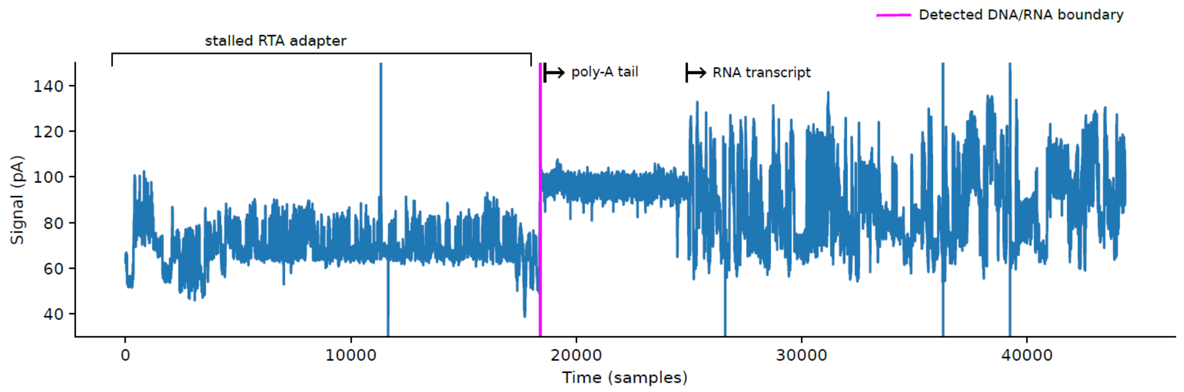

(a) Case 1

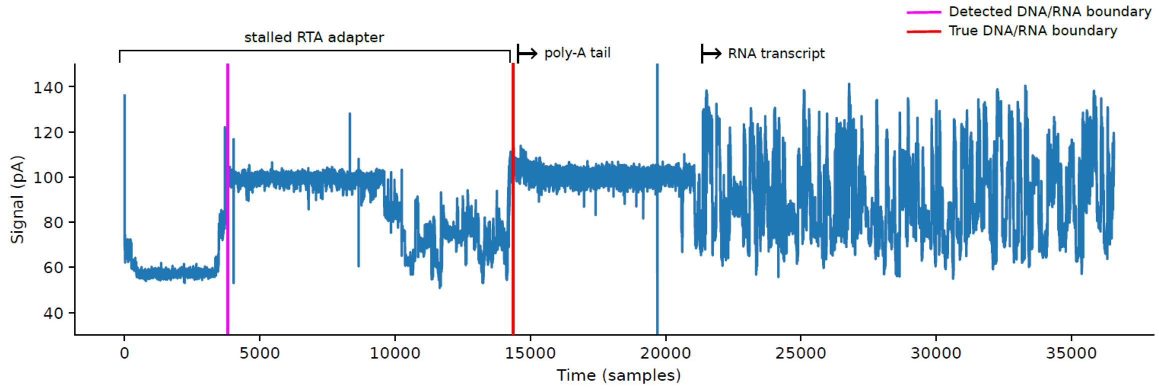

(b) Case 2

Supplementary Figure 2. Examples of noise class signal instances. (a) Stall in adapter signal leading to potential mis-segmentation. (b) Stall in the beginning of the adapter leading to mis-identification of DNA/RNA boundary.

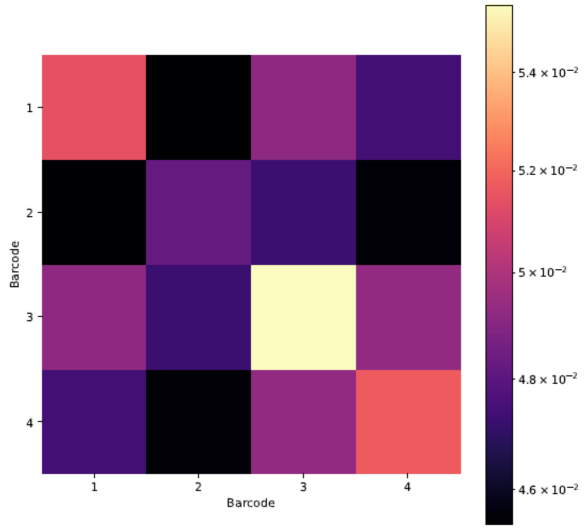

Supplementary Figure 3. The kernel matrix used to train the WDX4 model, with values averaged per class pair, shows the separation between barcode classes.

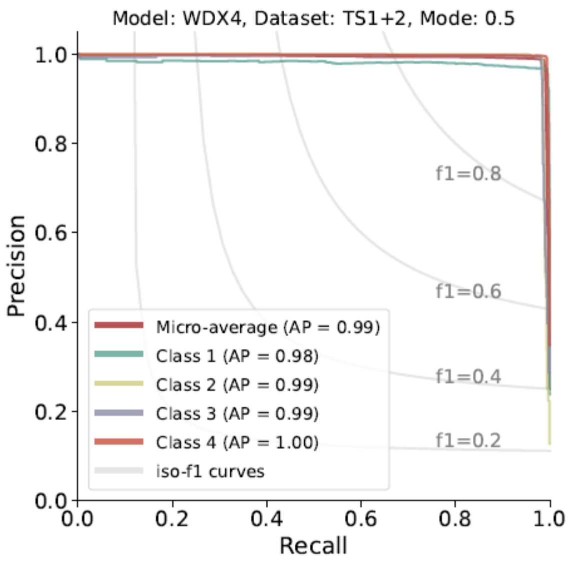

Supplementary Figure 4. Precision-recall curves of individual WDX-RTA barcodes used in WDX4 model. Evaluated on dataset 3–6 at a confidence cutoff of 0.5.

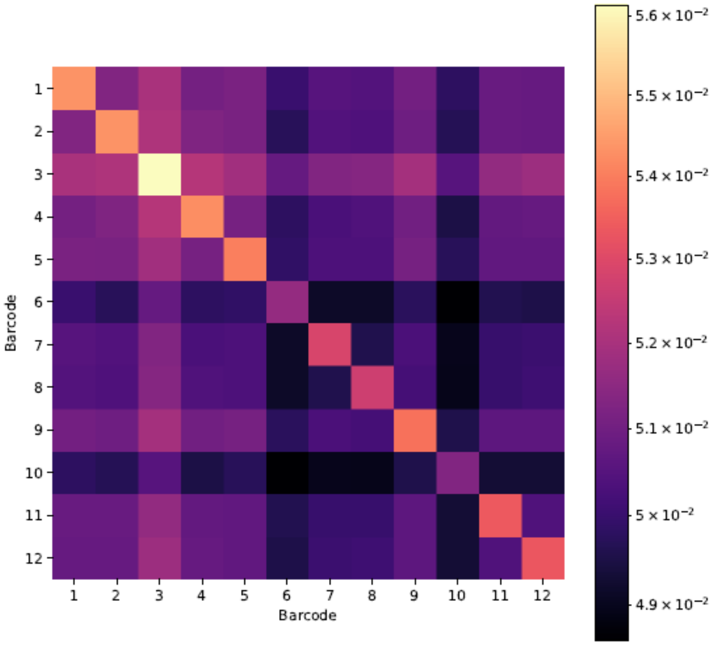

Supplementary Figure 5. The kernel matrix used to train the WDX12 model, with values averaged per class pair, shows the separation between barcode classes.

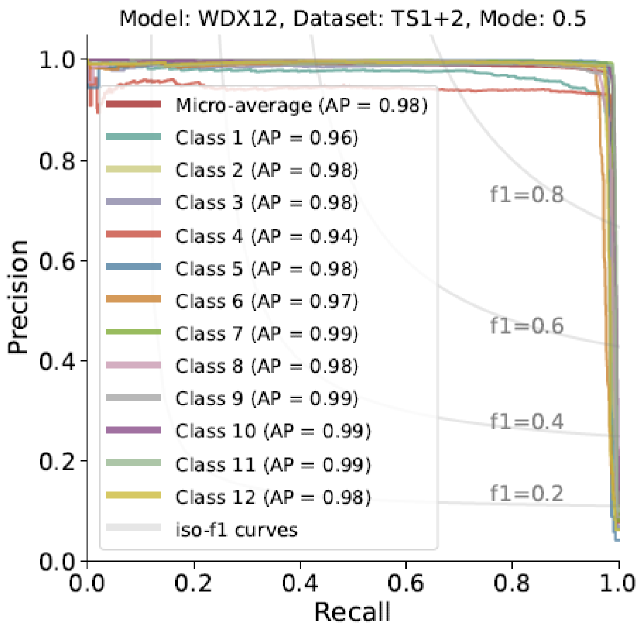

Supplementary Figure 6. Precision-recall curves of individual WDX-RTA barcodes used in WDX12 model. Evaluated on dataset 3–6 at a confidence cutoff of 0.5.

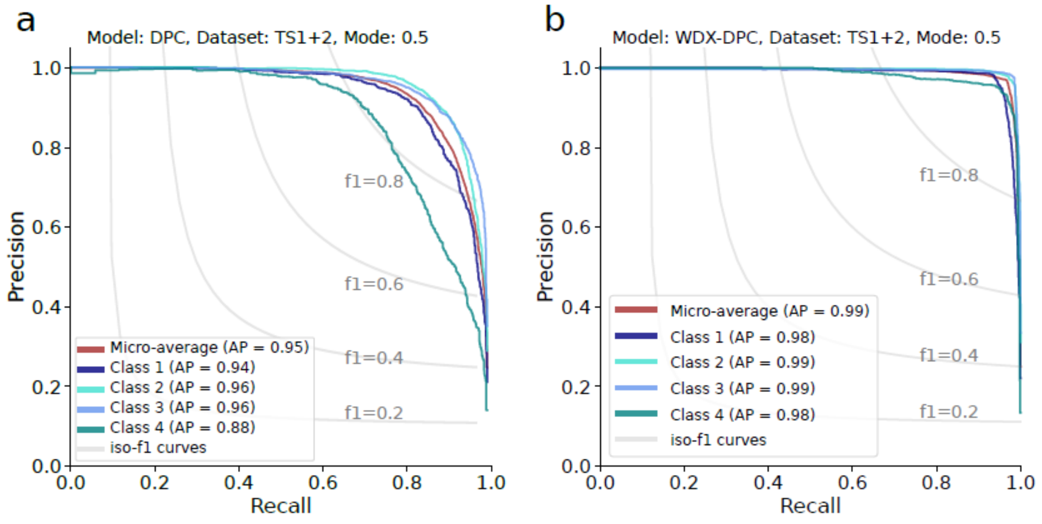

Supplementary Figure 7. Precision-recall curves comparing two methods. Panel a is for DeePlexiCon and panel b is for WDX-DPC. Evaluated on dataset 1–2 at a confidence cutoff of 0.5. Both models were trained on the same data (provided by the DeePlexiCon authors), and both evaluated on datasets 1 and 2. Left: Performance of published DeePlexiCon model. Right: Performance of WarpDemuX model.

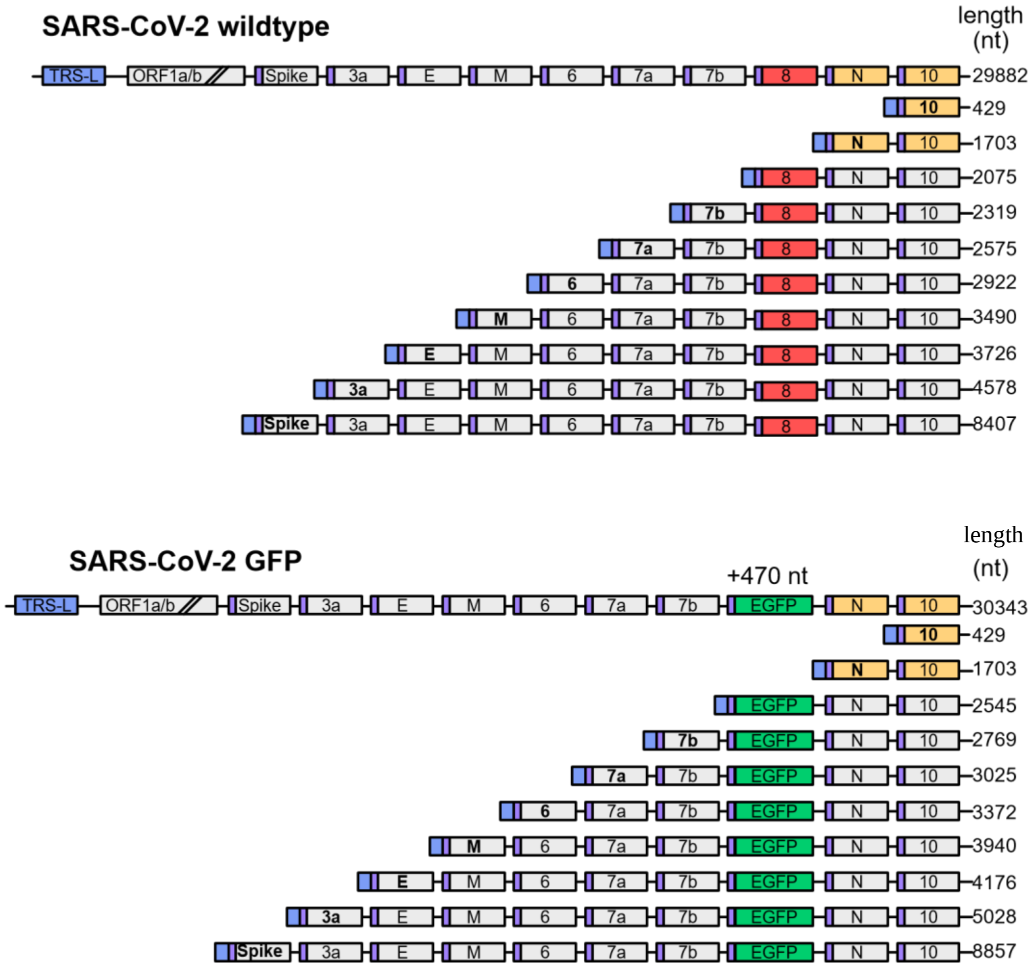

Supplementary Figure 8. Schematic of viral RNAs generated during infection. Upper panel is for the SARS2-CoV-2 wildtype and lower panel is for GFP-expressing variant (lower). TRS-L sequence is marked in light blue, the expressed isoform for subgenomic RNAs is boldened. The two isoforms that are not distinguishable between the wildtype and GFP variant are colored in yellow.

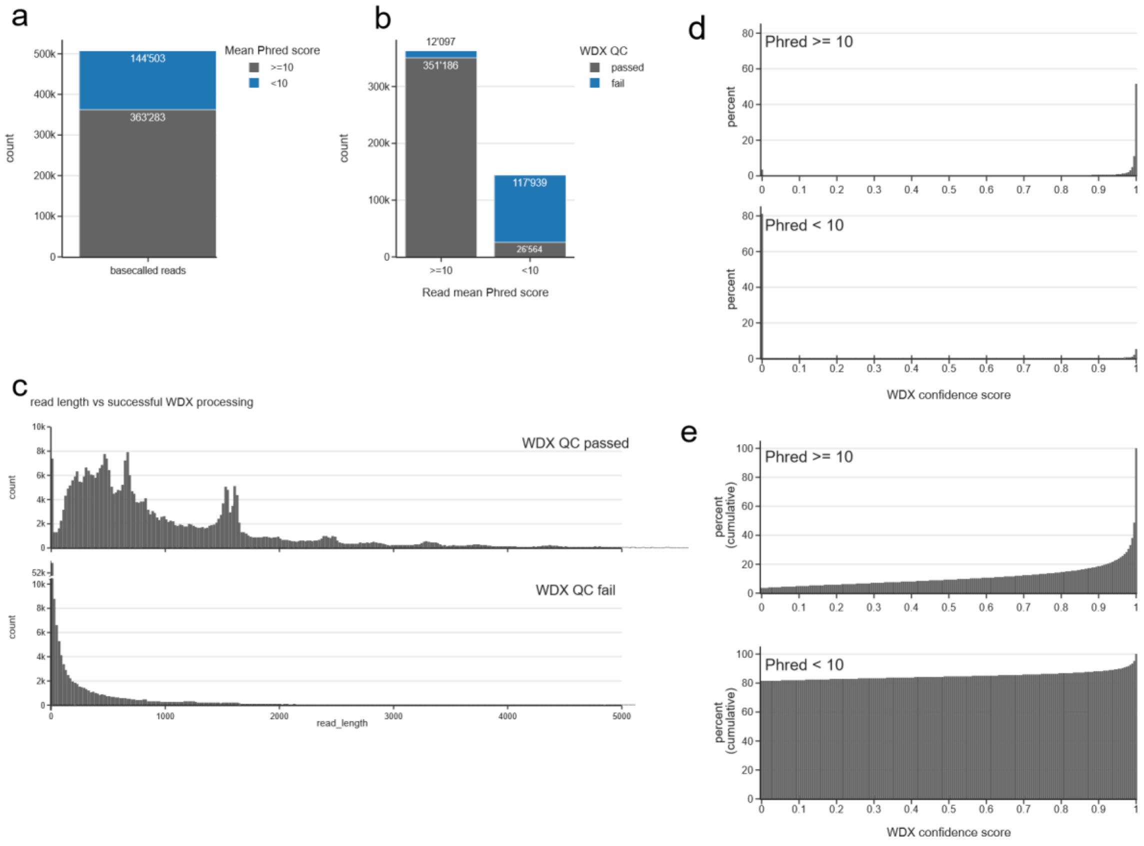

Supplementary Figure 9. Run statistics for the multiplexed SARS-CoV-2 sequencing run. (a) Total number of basecalled reads, split by mean read Phred score 10. (b) Number of reads passing WarpDemuX quality checks (poly(A) detection, signal quality checks) separated by mean read Phred qscore of 10. (c) Read length distribution of reads passing or failing WarpDemuX QC. (d) WarpDemux confidence score distribution for reads passing WDX QC separated by read mean qscore of 10. (e) Cumulative WarpDemuX confidence score distribution for reads passing WDX QC separated by read mean qscore of 10.

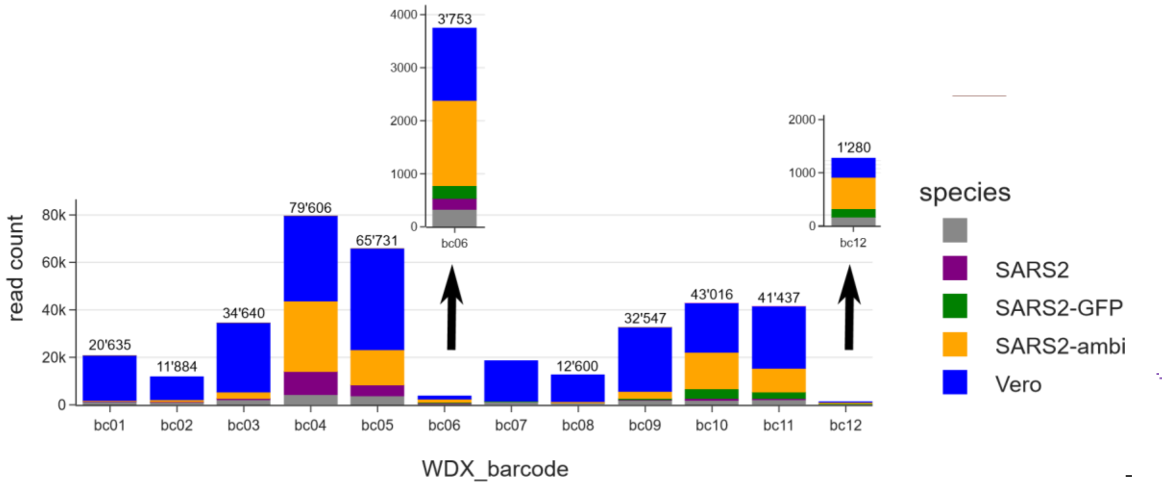

Supplementary Figure 10. Run statistics after WDX demultiplexing (without WDX confidence threshold). Number of reads classified per barcode at a WDX confidence score cutoff of 0, colored by species. The numbers describe the total read count per barcode.

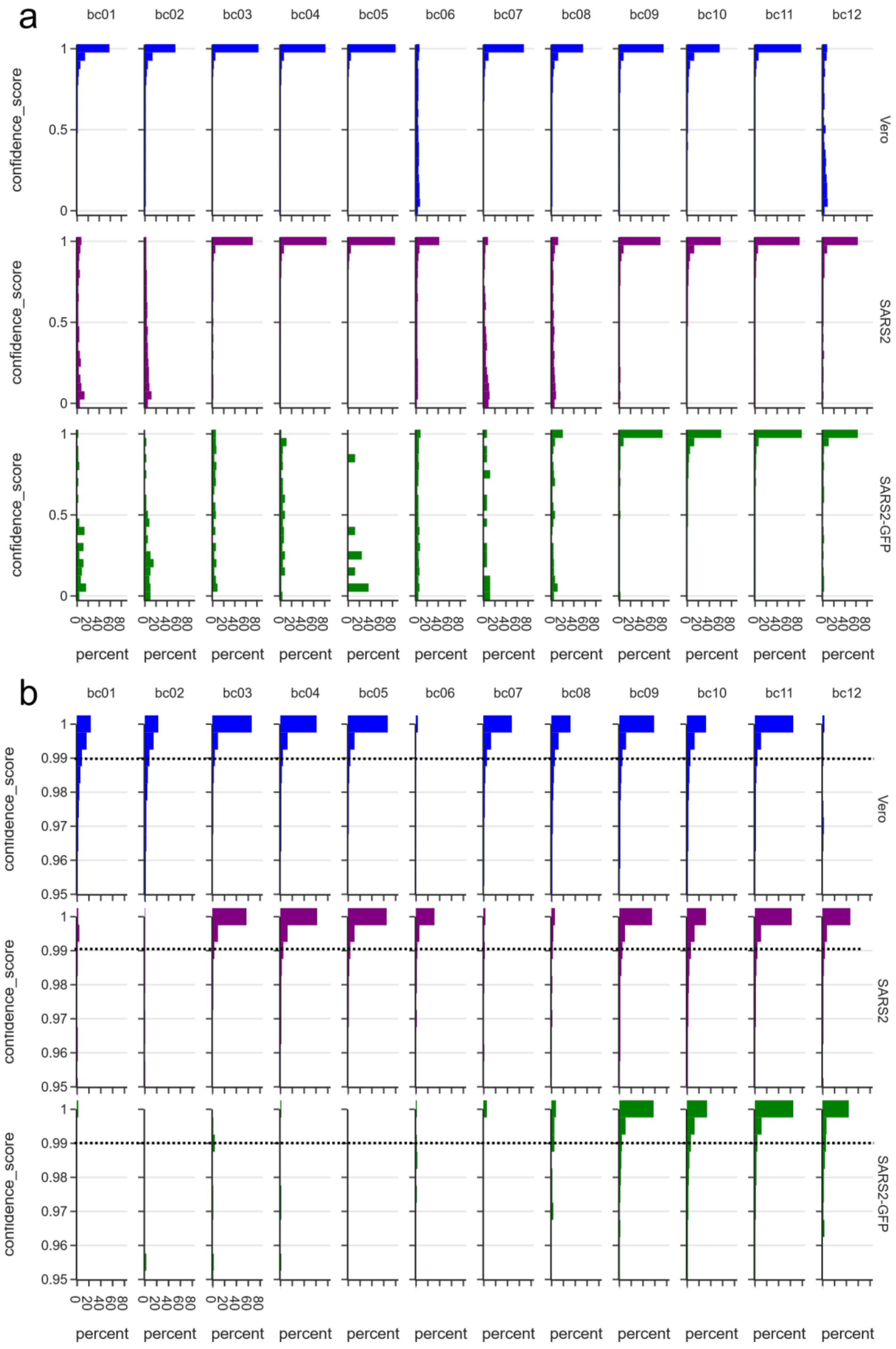

Supplementary Figure 11. WarpDemuX confidence threshold distribution per barcode and species. (a) WarpDemuX confidence score distribution (normalized as percent) per barcode (column) and species (row). (b) Zoom of (a) with higher resolution for confidence scores between 0.95 and 1.

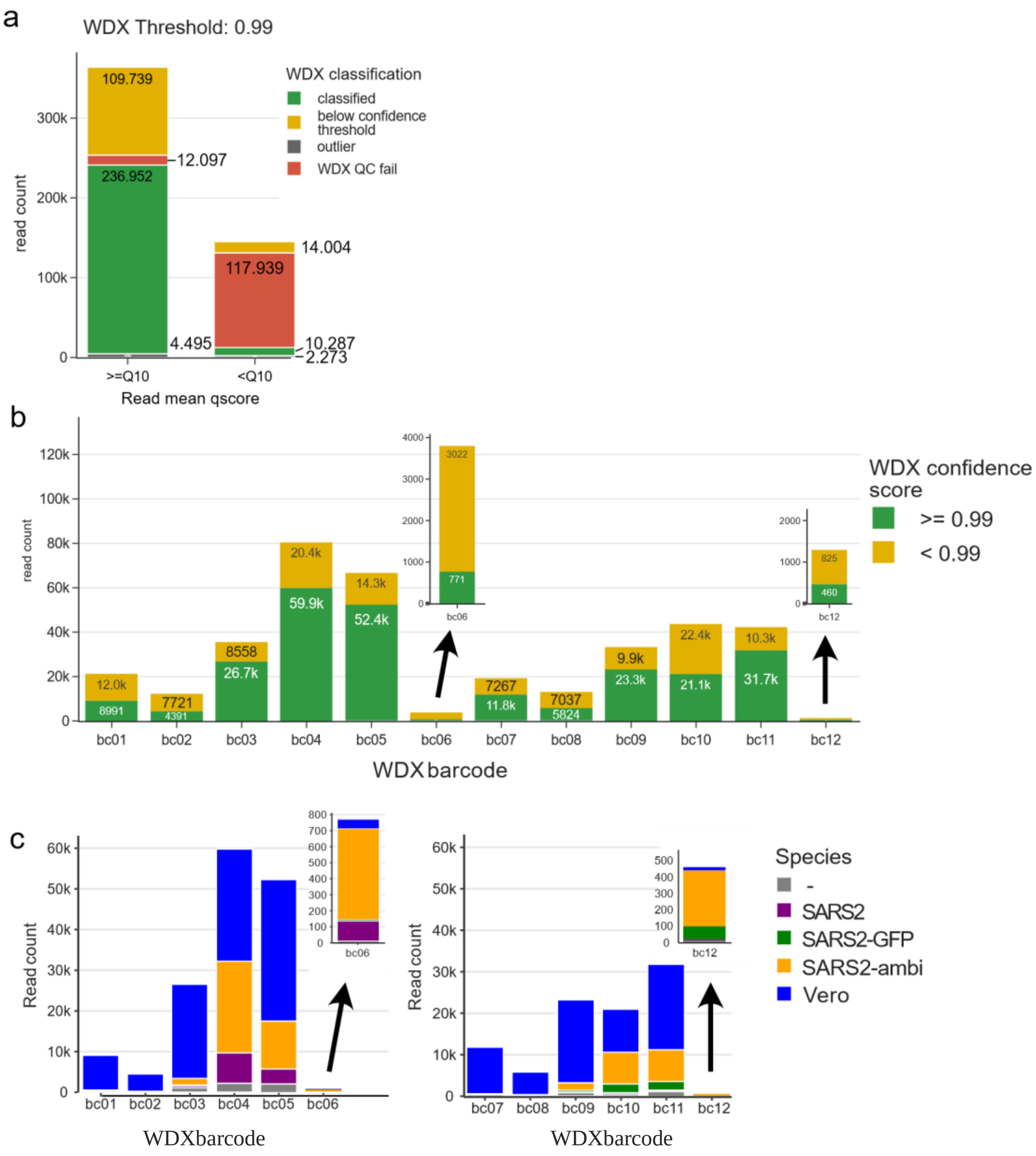

Supplementary Figure 12. WarpDemuX confidence threshold of 0.99. (a) Number of reads passing a WarpDemuX confidence threshold of 0.99 by read mean Phred score. (b) Number of reads per barcode passing the 0.99 confidence threshold. (c) Total number of reads aligning uniquely to the genome of a species per barcode at a WDX confidence threshold of 0.99.

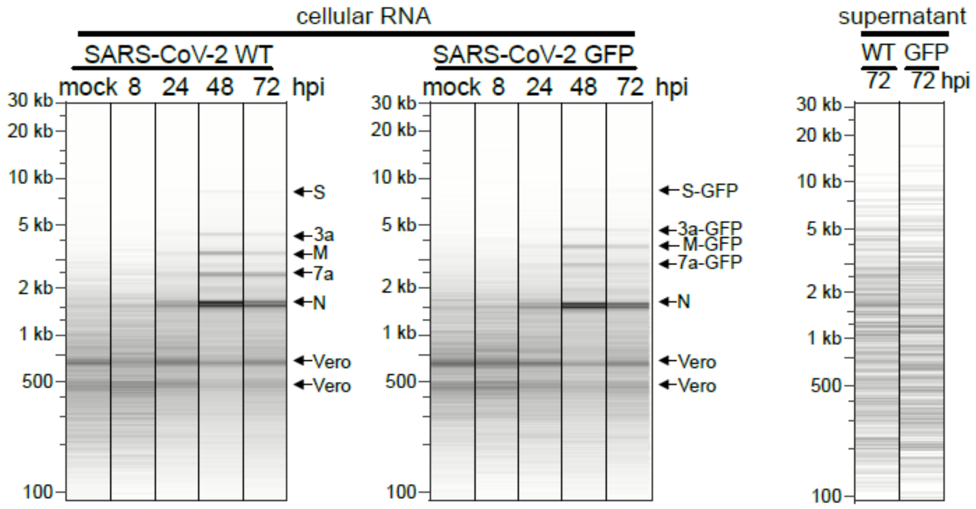

Supplementary Figure 13. Virtual gel of demultiplexed samples. Linear length scale, normalized per sample. The annotations highlight bands corresponding to SARS-CoV-2 isoforms, with the GFP variant offset by around 500 nt above the WT variant.

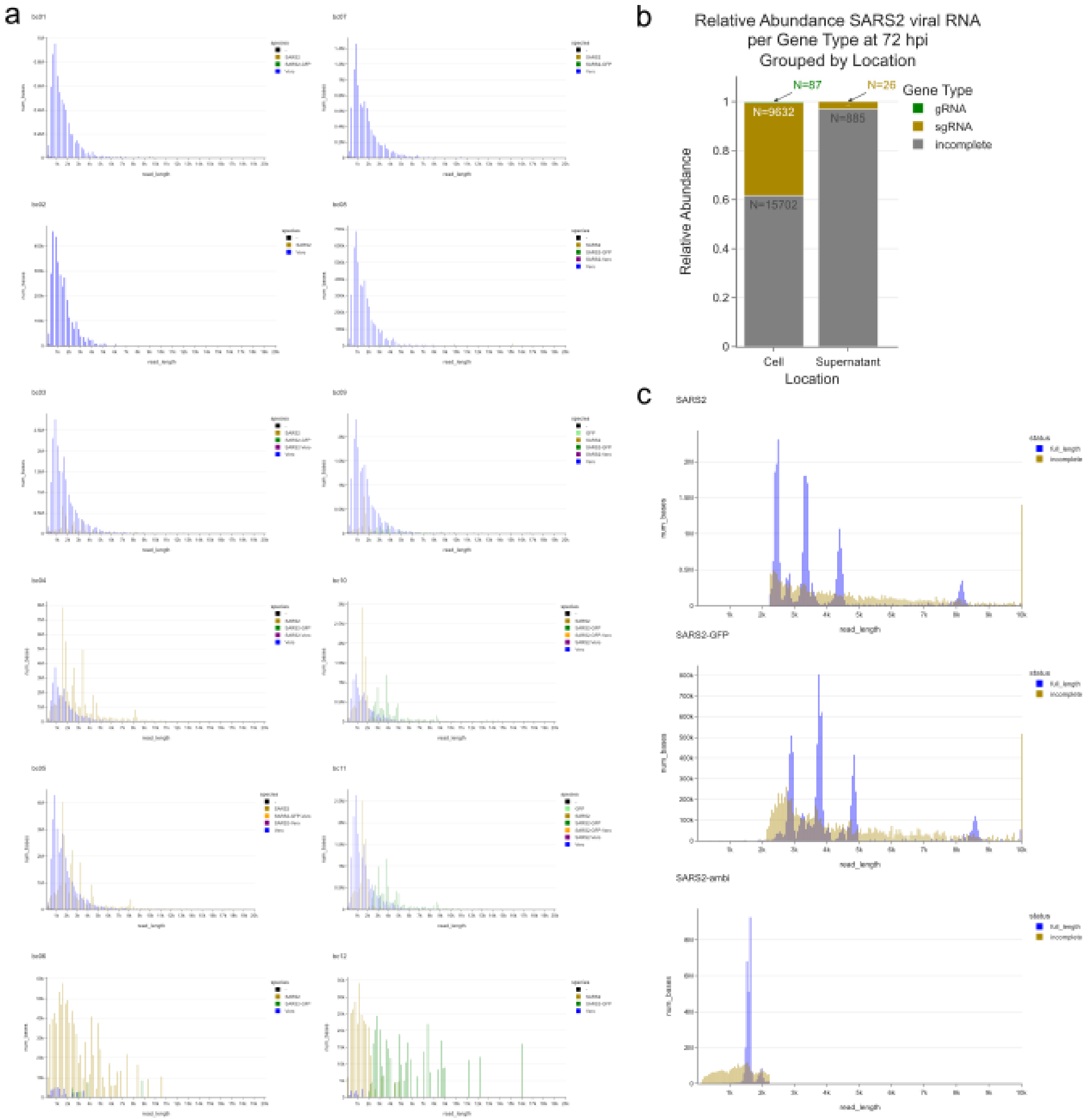

Supplementary Figure 14. Read length distribution per barcode and species. Classification of SARS2 reads according to presence of TRS-L sequence. (a) Histogram of number of bases by read length for reads mapping to the different species (not split by full length). (b) Relative abundance of SARS-CoV-2 reads for which a TRS-L sequence was detected separated by location of RNA. (c) Read length distributions for full length and incomplete reads aligning to either SARS2-WT (top), SARS2-GFP (middle) or ambiguously to SARS2 (bottom).

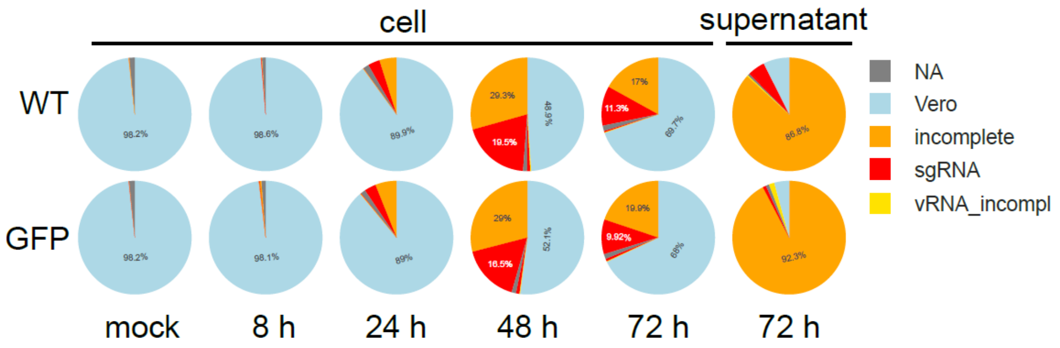

Supplementary Figure 15. Pie charts of relative abundance of reads aligning to Vero and SARS2, split by incomplete and full length transcripts for SARS2.

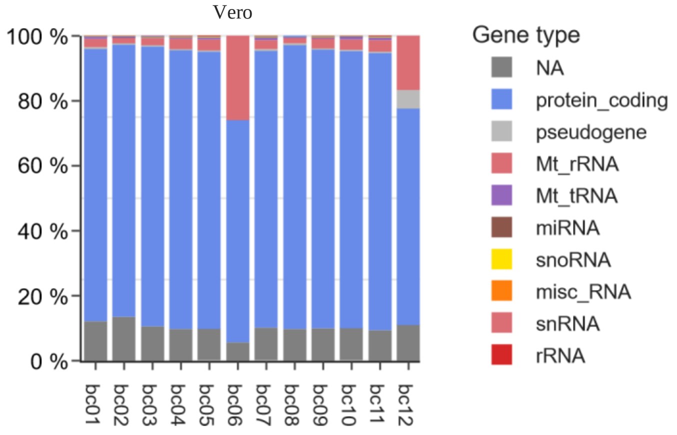

Supplementary Figure 16. Relative bar charts per sample of gene assignments (generated by isoquant) for reads aligning to the Vero genome.

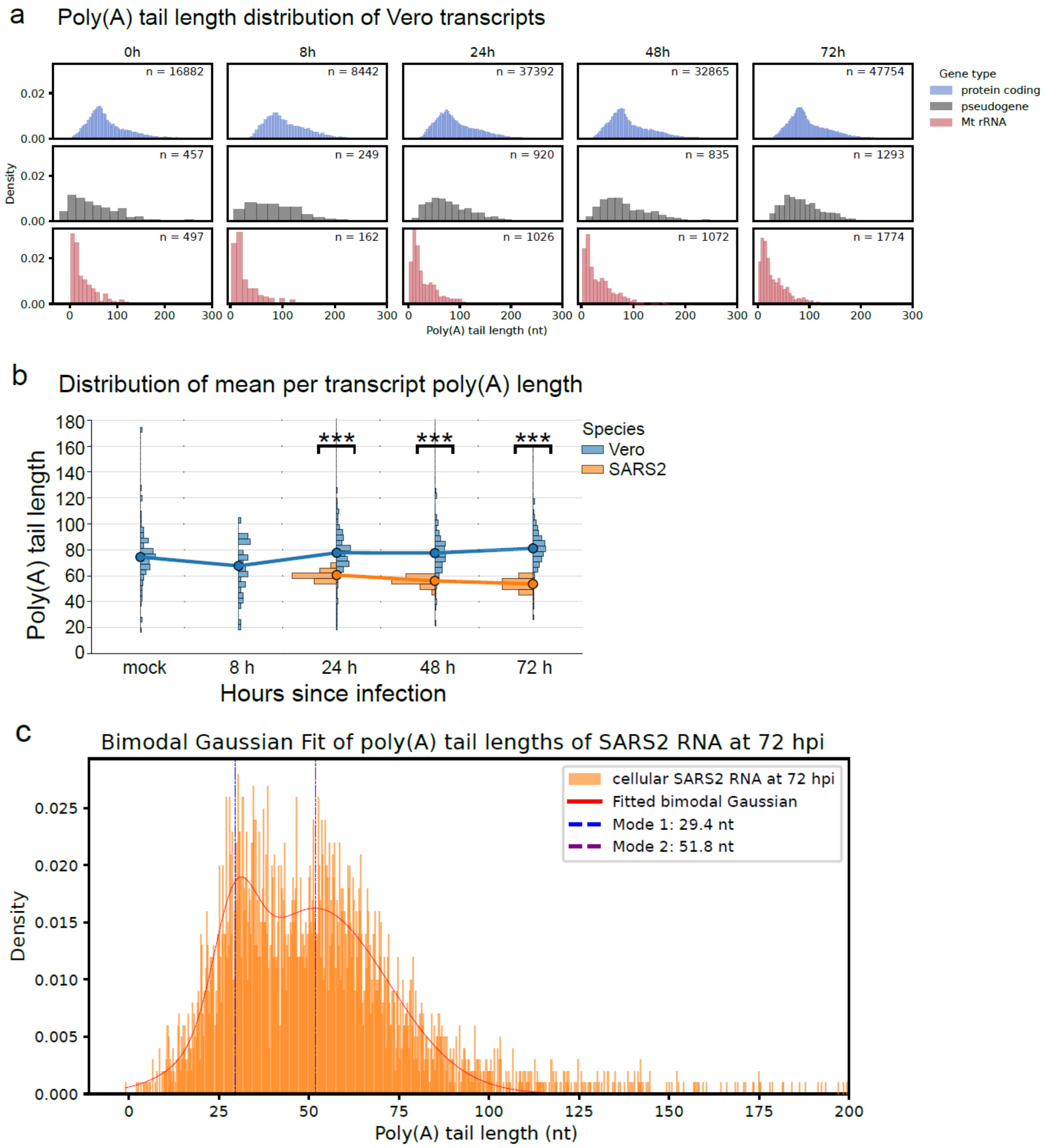

Supplementary Figure 17. Poly(A) tail length distribution statistics. (a) Poly(A) tail length distributions of Vero transcripts by gene type (biomart annotation). (b) Poly(A) tail length distribution per transcript. For each transcript with coverage of at least 20 the mean poly(A) tail length was calculated. (c) Bimodal Gaussian fit of the poly(A) tail length distribution of SARS2 reads at 72 hpi to determine the modal poly(A) tail length for the two distributions.

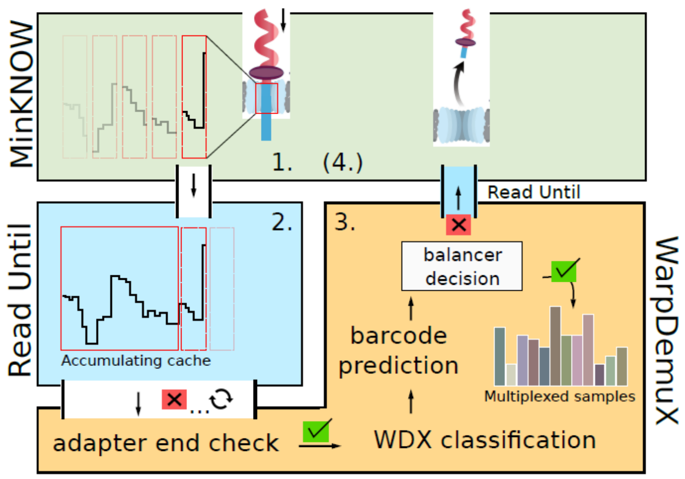

Supplementary Figure 18. Overview software design WarpDemuX adaptive sampling. (1) The sequencer (MinKNOW) streams data in chunks of a fixed size. (2) ReadUntil communicates with the sequencer API and accumulates the read data in an 'Accumulating cache'. (3) WarpDemuX watches the accumulating cache for the start of a poly(A) ('adapter end check'). When found, the adapter signal is send off to the WarpDemuX classification pipeline. Based on the predicted barcode label and the current sequencing distribution over multiplexed samples, a balancing decision is made (retain/reject). To reject a read (4), WarpDemuX sends an 'unblock' request to MinKNONW, via ReadUntil.

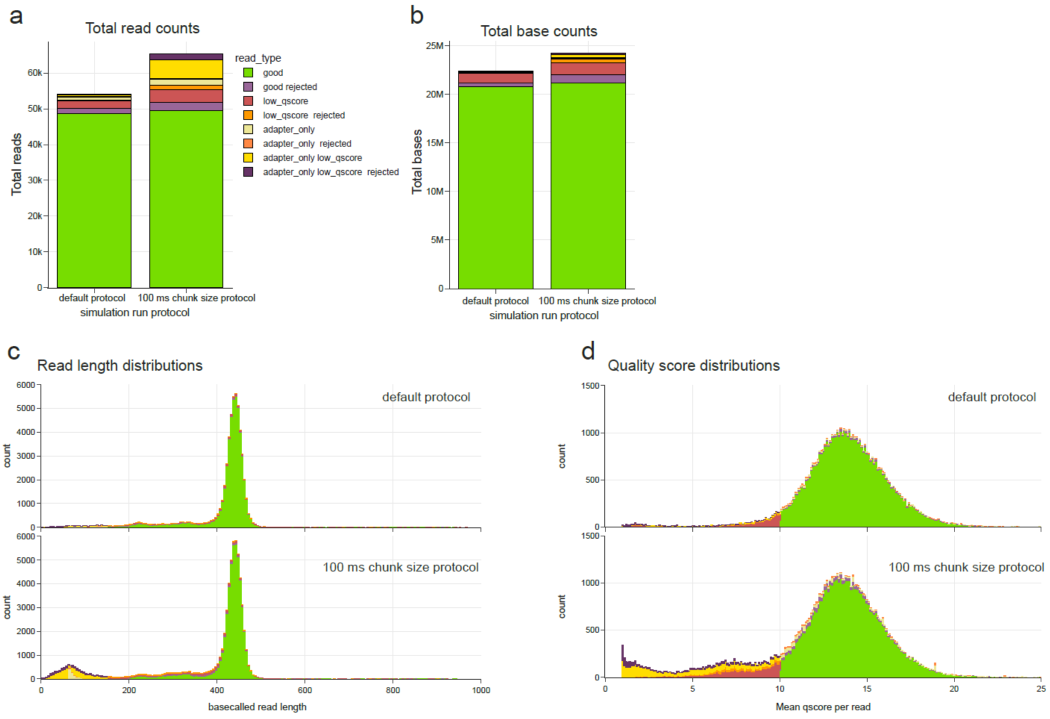

Supplementary Figure 19. Comparison of reduced chunk size sequencing protocol against the default protocol. The comparative data was generated by replaying a sequencing run recorded with the default protocol with both the default and our decreased chunk size protocol. (a) Total number of generated reads per protocol (low_qscore: mean qscore <10, adapter_only: read length <150 nt) (b) Total number of generated bases per protocol. (c) Read length distribution of basecalled reads for both default and decreased chunk size protocol, colored according to legend of (a). (d) Read mean qscore distribution colored according to legend of (a).

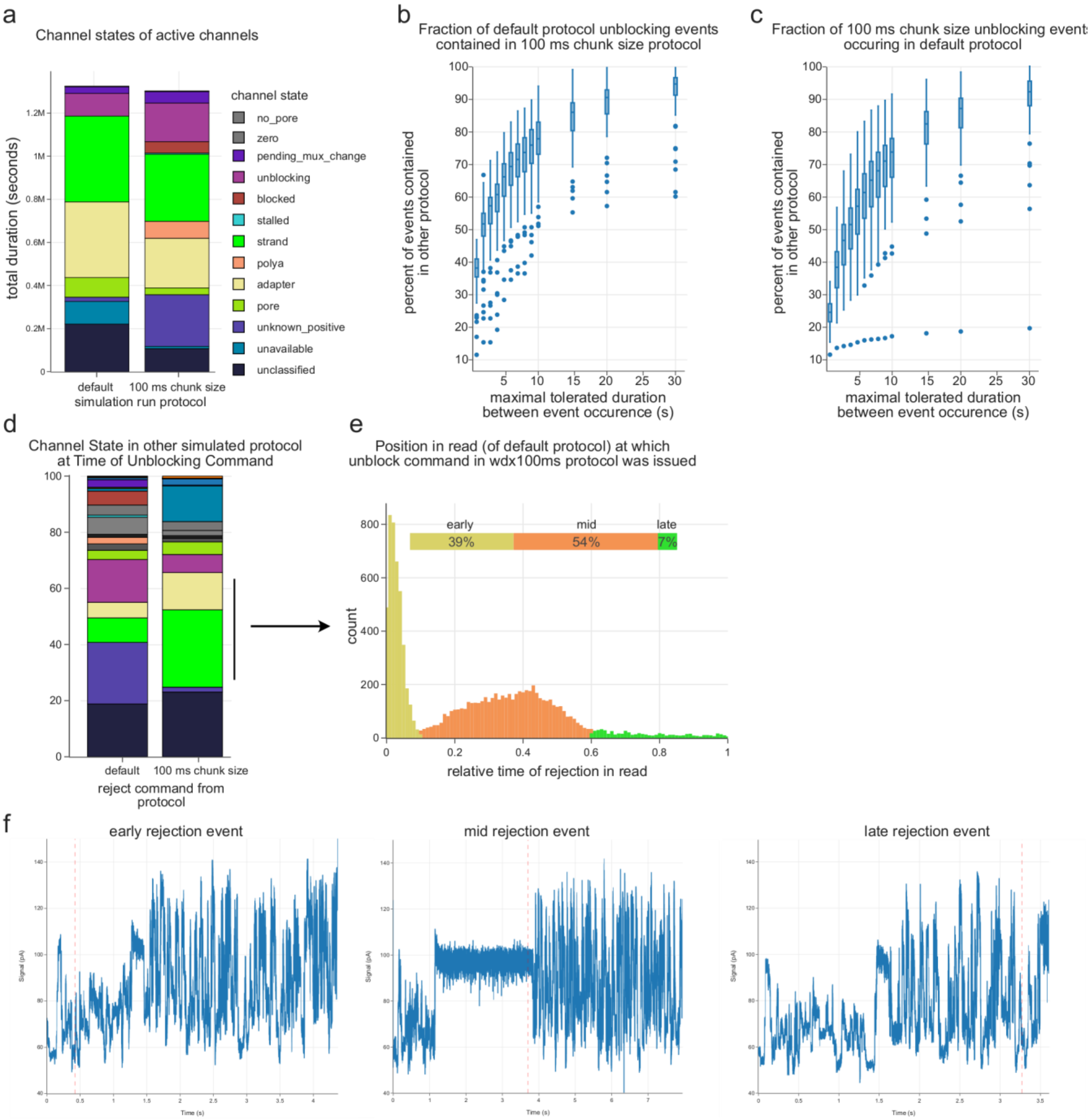

Supplementary Figure 20. Comparison of channel states of different sequencing protocols. (a) Distribution of channels state durations for each protocol. The decreased chunk size protocol has additional channel states as defined in RNA2_seq_WDX_live_channel_states.toml. (b) Fraction of unblock events issued by the default protocol contained within a given duration window of reject commands issued by the 100 ms chunk size protocol. Each active channel is one data point. Horizontal lines indicate the median, boxes denote the inter-quartile range (IQR) and whiskers extend to 1.5x IQR. (c) Fraction of unblock events issued by the 100 ms chunk size protocol contained within a given duration window of reject commands issued by the default protocol. Each active channel is one data point. (d) Channel state of the comparison protocol during the issuing of an unblock command, colored according to (a). (e) Distribution of position of 100 ms chunk size reject command in reads written out by the default protocol. (f) Example reads identified in (e) for each of the three unblock event types.

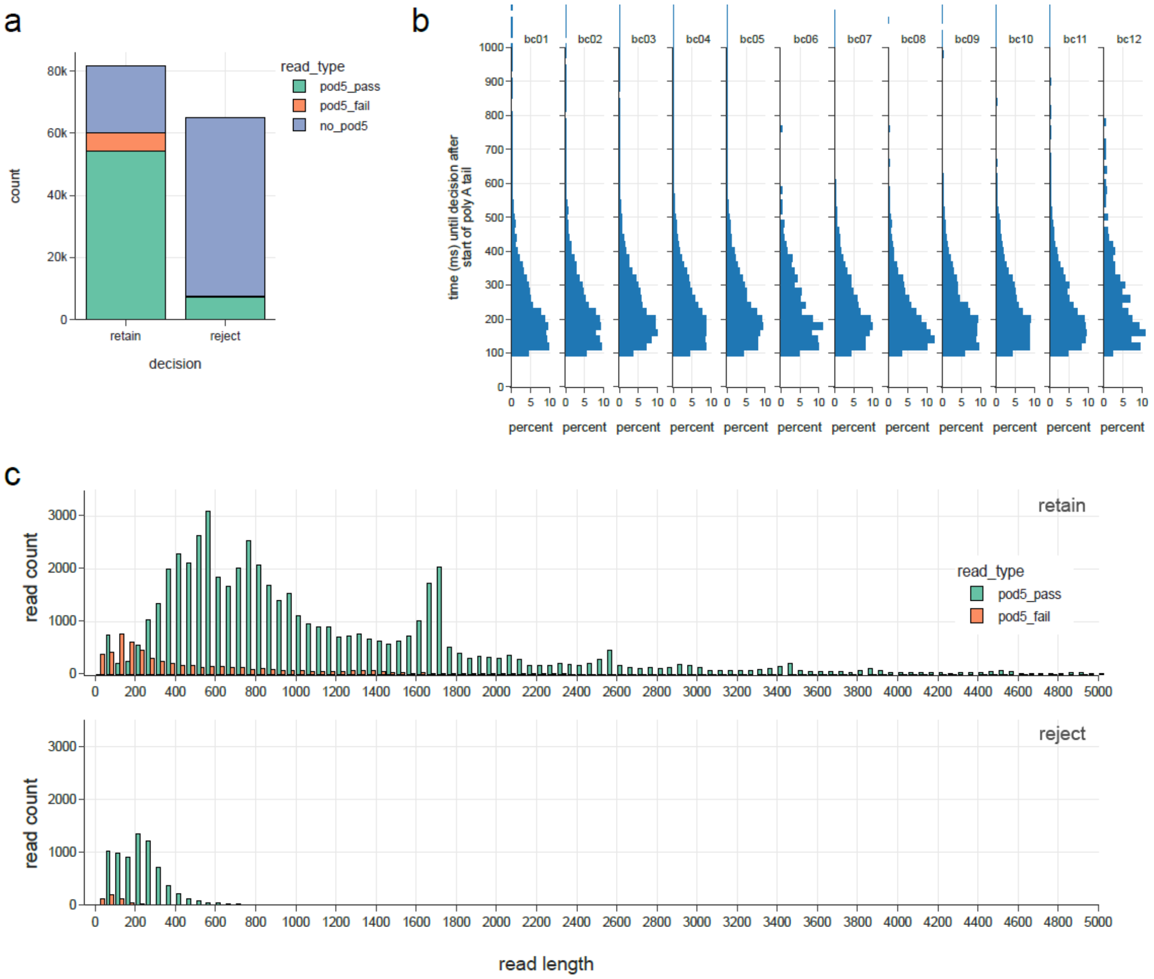

Supplementary Figure 21. Latency of live WarpDemuX balancing. (a) Reads with reject decisions are most of the time too short to be written into pod5 files. (b) The duration between the start of the poly(A) sequence and the reject decision is constant between barcodes. (c) Read length distribution (after basecalling) for reads that are written into pod5 files, separated by decision and qscore filter.

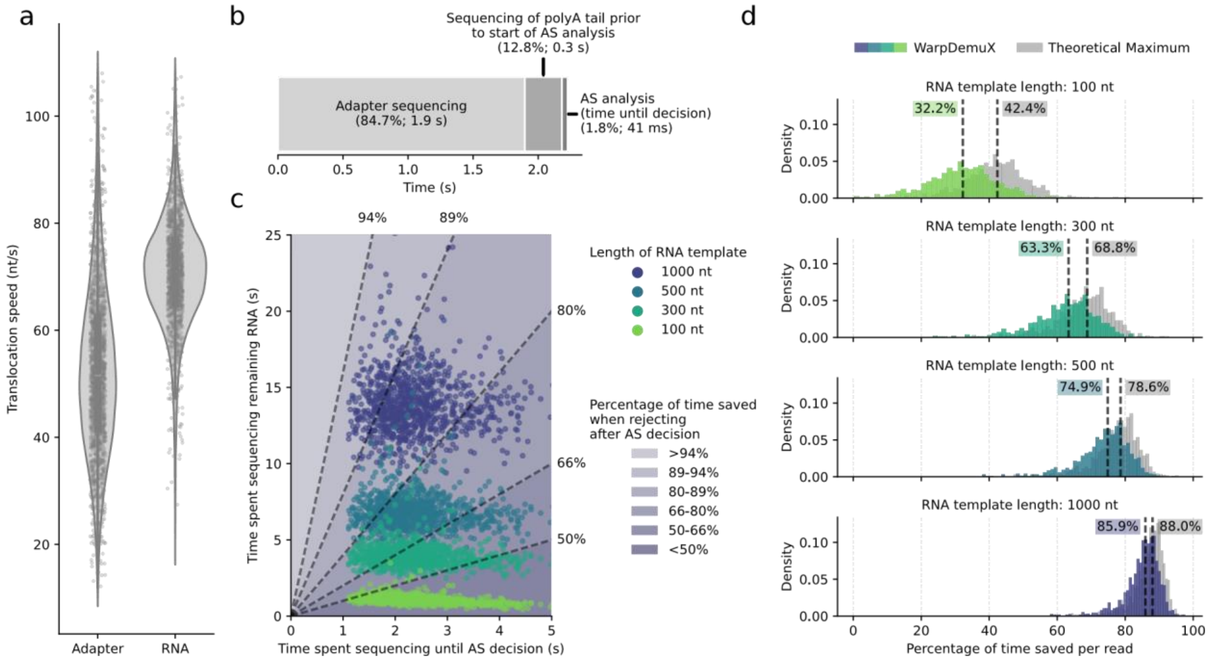

Supplementary Figure 22. Analysis of WarpDemuX adaptive sampling efficiency. (a) Distribution of translocation speeds showing distinct rates for adapter and RNA regions. (b) Temporal breakdown of adaptive sampling (AS) process from molecule entry to decision, showing contributions from adapter sequencing, WarpDemuX latency, and analysis time. Annotated values represent median estimates. (c) Estimated time savings for rejected reads across different template lengths, demonstrating >50% time savings for 90% of reads with templates >300 nt, increasing to >65% and >80% for 500 nt and 1000 nt templates respectively. (d) Comparison of actual WarpDemuX performance against theoretical maximum enrichment (zero latency case), showing near-optimal performance for templates >300 nt despite real-world constraints.

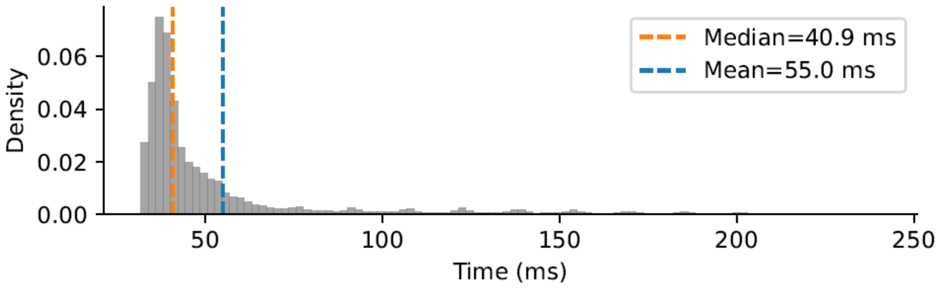

Supplementary Figure 23. Distribution of WarpDemuX adaptive sampling analysis times.

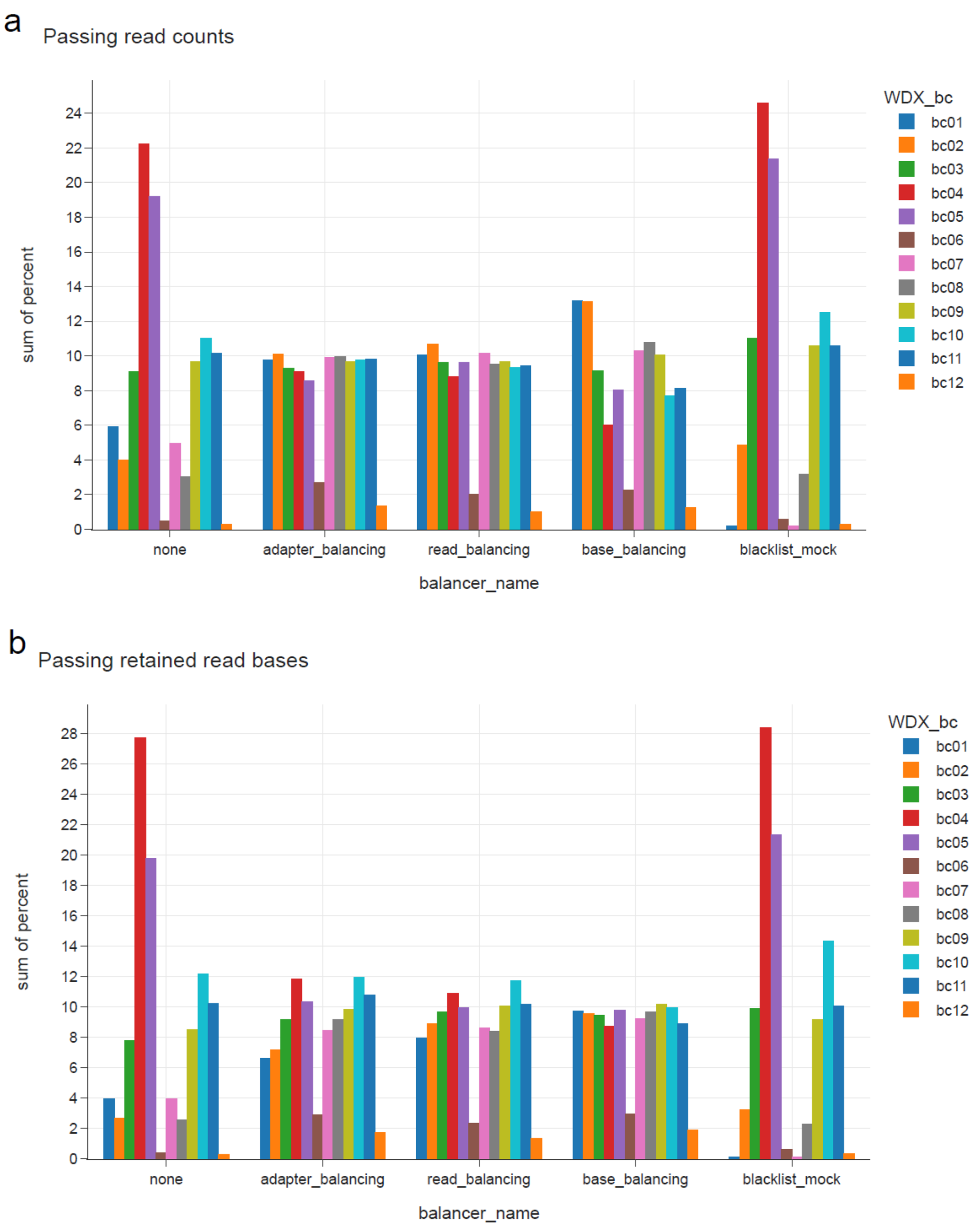

Supplementary Figure 24. Balancer strategies. Balancer name 'none' indicates no adaptive-sampling. 'adapter_balancing' balances based on relative counts of classified adapters. 'read_balancing' and 'base_balancing' combine relative counts with information from the pod5 files created during sequencing to correct relative counts (discarding adapter-only reads). 'base_balancing' takes into account the estimated bases sequenced per barcode based on the read length in the pod5 file. In 'blacklist_mock', barcode 1 and 7 were blacklisted. Passing reads refer to non-rejected reads subsequently written out to the pod5_pass sequencing output folder. (a) Relative abundance of reads for each barcode when different balancer strategies are applied. (b) Relative abundance of bases for each barcode when different balancer strategies are applied.

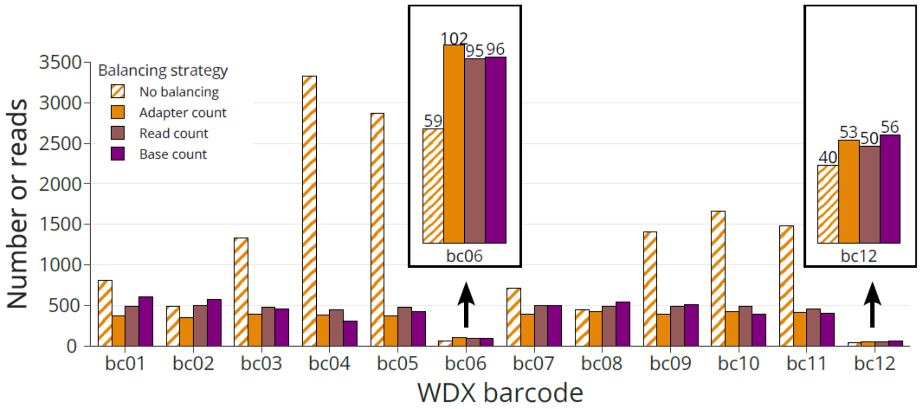

Supplementary Figure 25. Bar plot showing total number of reads recovered with and without balancing for different balancing strategies (40 channels each). Inset shows enrichment for low abundance samples. No balancing indicates no adaptivesampling, all reads are accepted. Adapter count balances based on relative counts of classified adapters. Read counts and Base counts strategies combine the relative barcode counts during adaptive sampling with information from the pod5 files created during sequencing to correct relative counts for adapter-only reads (discarding adapter-only reads). Base counts takes into account the estimated bases sequenced per barcode based on thed read lengths in the pod5 file.

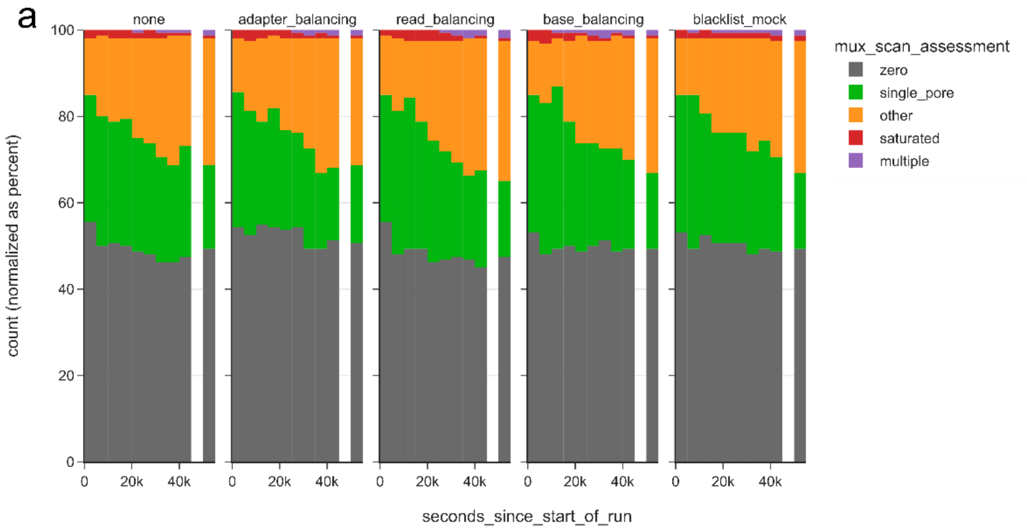

Supplementary Figure 26. Pore lifetime with different balancer strategies. Visualization of pore scan results during the live adaptive sampling sequencing run. Balancer name 'none' indicates no adaptive-sampling. 'adapter_balancing' balances based on relative counts of classified adapters. 'read_balancing' and 'base_balancing' combine relative counts with information from the pod5 files created during sequencing to correct relative counts (discarding adapter-only reads). 'base_balancing' takes into account the estimated bases sequenced per barcode based on the read length in the pod5 file. In 'blacklist_mock', barcode 1 and 7 were blacklisted.

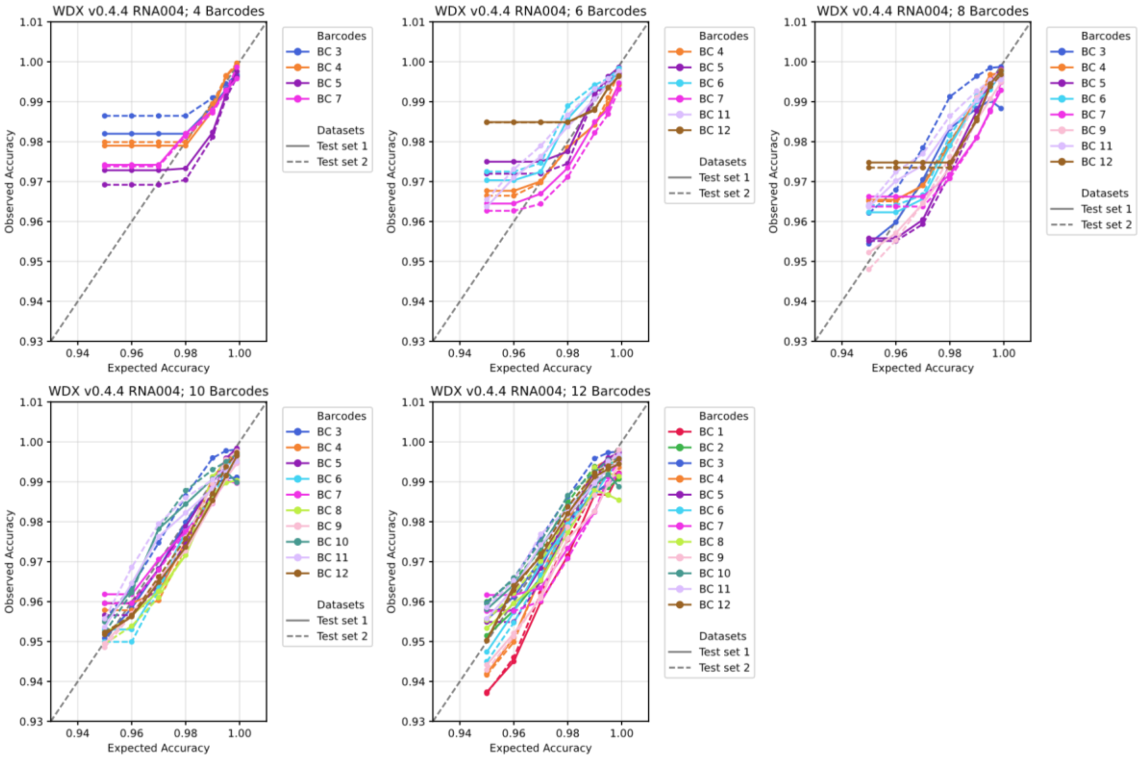

Supplementary Figure 27. Calibration performance of WDX SQK-RNA004 models across varying barcode counts (4–12). Models were evaluated on two independent test sets, comparing observed vs. target accuracies at different Target Accuracy Modes (TAM: 95–99.9%). Solid and dashed lines represent test sets 1 and 2 respectively, while the diagonal gray line indicates perfect calibration. Consistent performance between test sets is demonstrated. For models and barcode combinations with baseline accuracy ≥95%, calibration at lower TAMs could only be achieved by reducing the model performance, while also reducing the yield. This is counterproductive and therefore not applied, resulting in observed accuracies that exceed the target at lower TAMs, effectively making the target accuracy a lower bound.

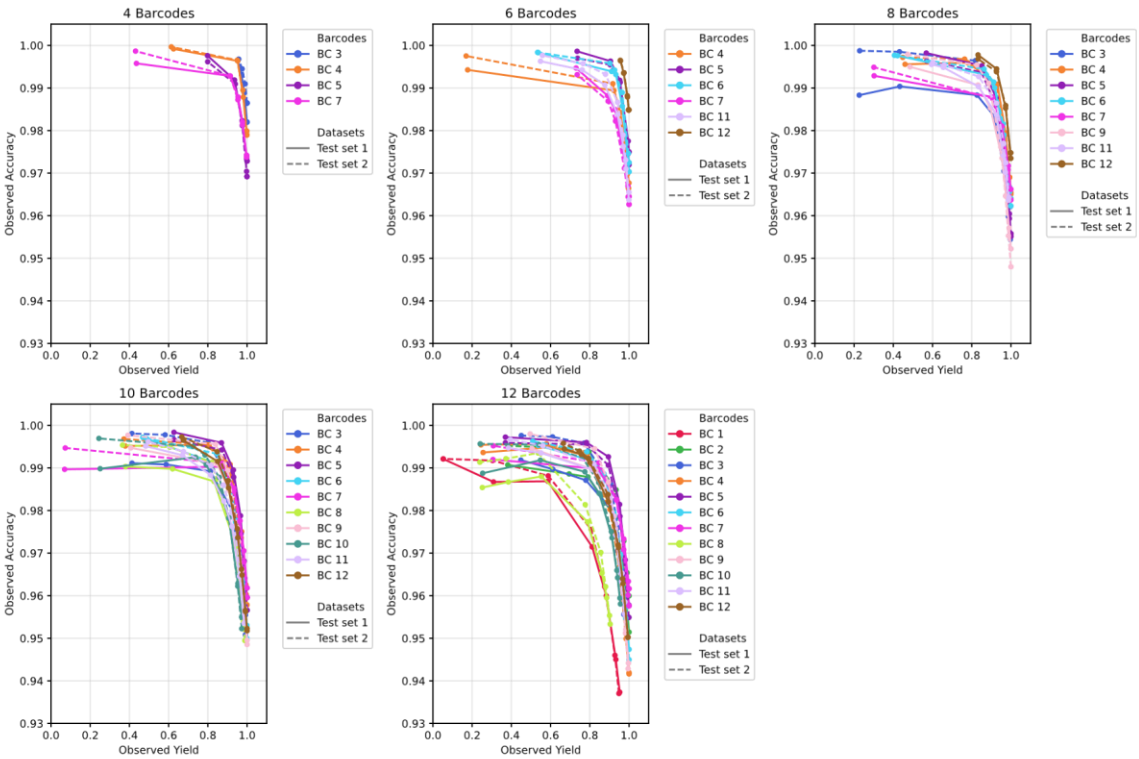

Supplementary Figure 28. Accuracy-yield trade-off curves for calibrated WDX SQK-RNA004 models with varying barcode counts (4–12) evaluated on two independent test sets. Each line represents the relationship between observed accuracy and yield (fraction of classified reads) for individual barcodes, with solid and dashed lines representing test sets 1 and 2 respectively. The plots demonstrate consistent performance between test sets and reveal barcode-specific variations in classification confidence. As the number of barcodes increases, achieving high accuracy (>99%) requires excluding a larger fraction of reads, illustrated by the leftward shift of the curves for higher barcode counts. This pattern aligns with the calibration results and highlights the inherent trade-off between classification complexity and model performance.

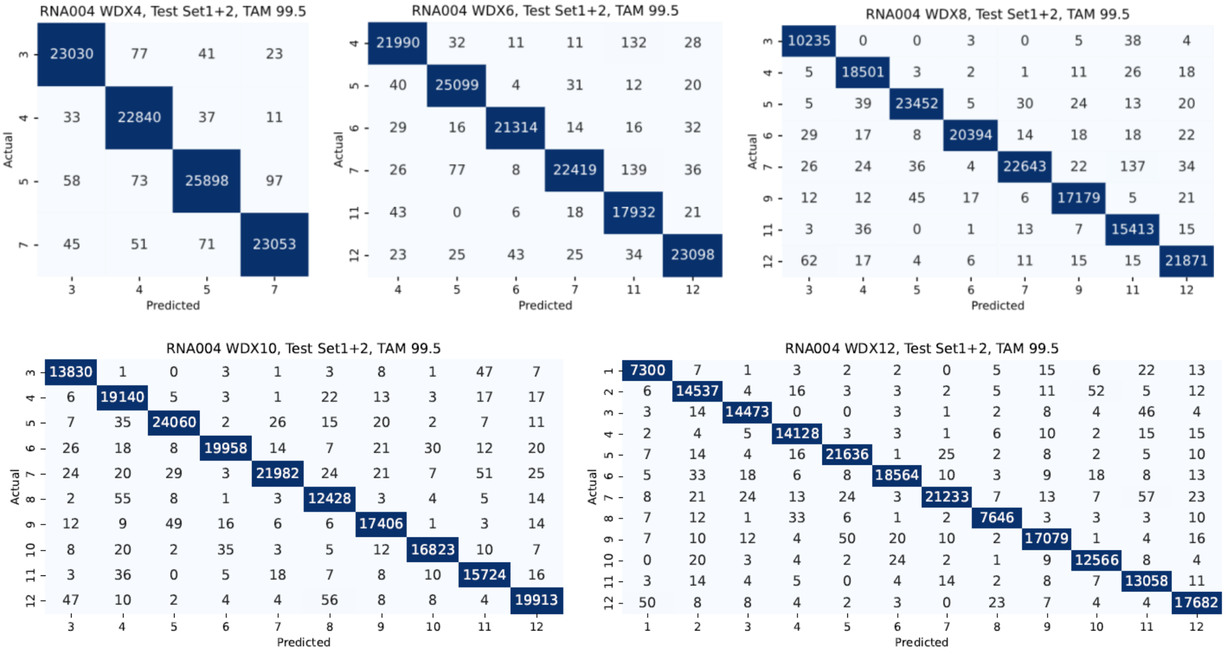

Supplementary Figure 29. Confusion matrices for calibrated WDX SQK-RNA004 models with varying barcode counts (4–12) at Target Accuracy Mode (TAM) 99.5% evaluated on Test set 1 and 2 combined.

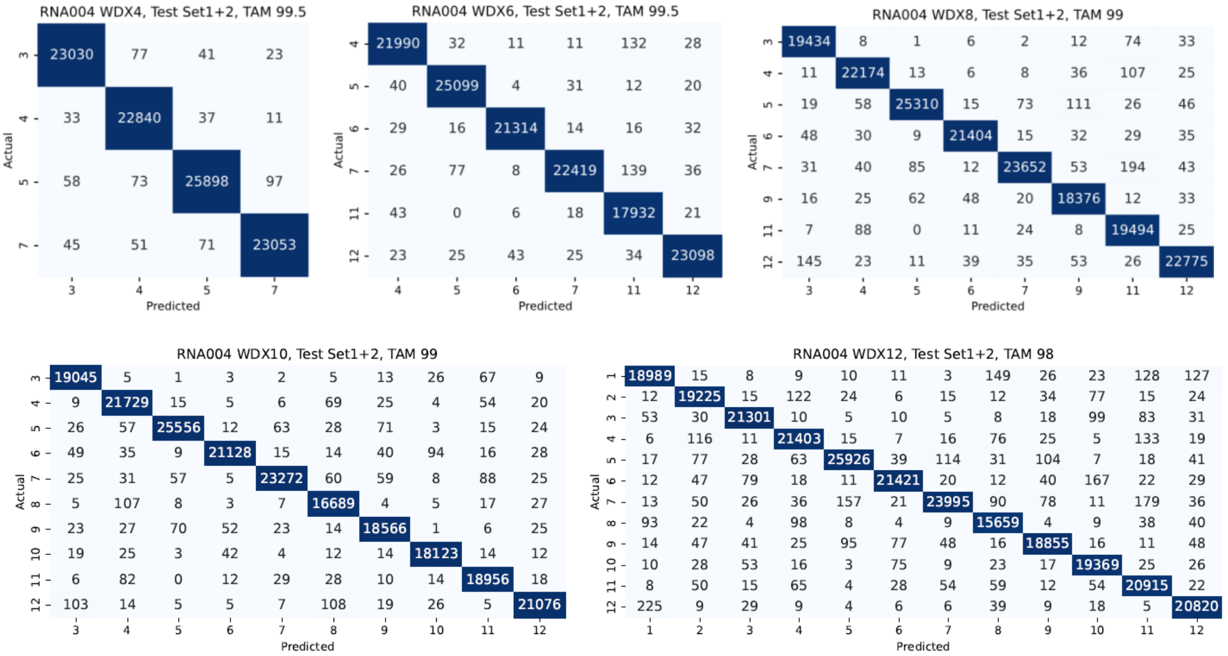

Supplementary Figure 30. Confusion matrices for calibrated WDX SQK-RNA004 models with varying barcode counts (4–12) at maximal Target Accuracy Mode (TAM) to achieve at least 85% yield per model (<15% Unclassified Reads), evaluated on Test set 1 and 2 combined. TAMs per model: WDX4: 0.995, WDX6: 0.995, WDX8: 0.99, WDX10: 0.99, WDX12: 0.98

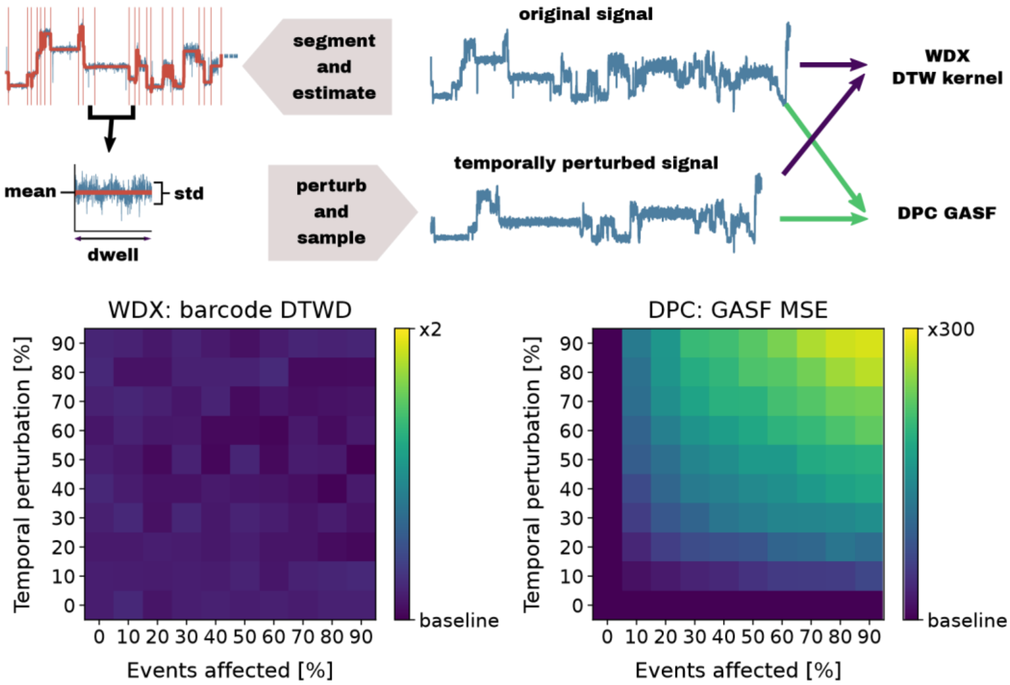

Supplementary Figure 31. Temporal perturbation analysis between DTWD and GASF. A comparative study illustrating the susceptibility of the GASF feature transformation to temporal perturbations, as opposed to the robustness exhibited by DTWD.

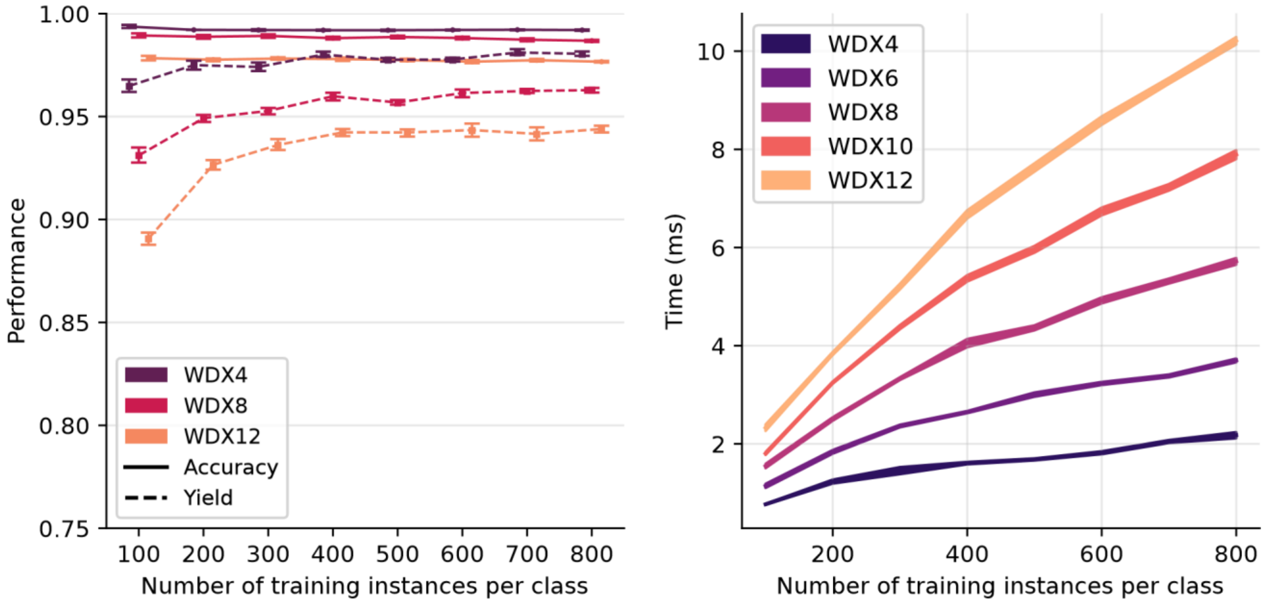

Supplementary Figure 32. Performance on validation set during model selection. Left: Performance and yield for increasing number of training instances per barcode, evaluated at confidence threshold of 0.5, shows that the gains of increasing number of training instances per barcode on the model's quality already dimish at small training set sizes. Values represent mean over 10-fold cross-validation with errorbars indicating 95% CI. Right: Classification time per read for increasing number of training instances per barcode. Shaded area reflects the average ±2std classification time per read, computed from 10 runs of 4000 reads on 8 CPU cores (11th Gen Intel(R) Core(TM) i7–1165G7 (2.80GHz) with 32GB memory).

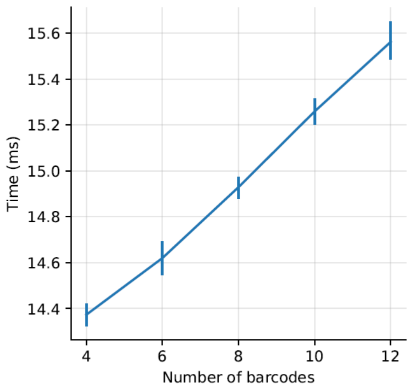

Supplementary Figure 33. Run time per read for WarpDemuX models increases linearly with the number of barcodes. Reported times reflect average time per read, computed from 10 runs of 4000 reads on 8 CPU cores (11th Gen Intel(R) Core(TM) i7–1165G7 (2.80GHz) with 32GB memory). Error bars span the mean ± one standard deviation.

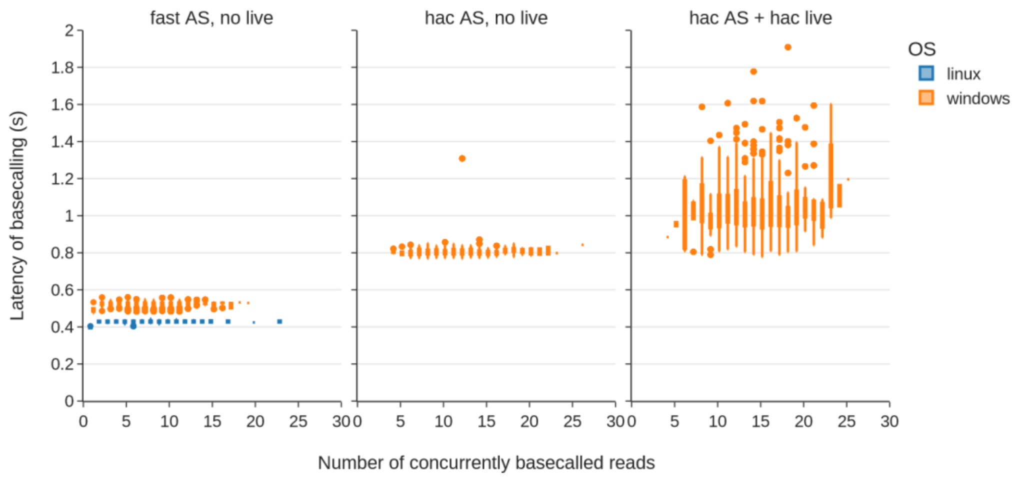

Supplementary Figure 34. Latency of basecalling for RNA adaptive sampling. Adaptive sampling (AS) at 0.1s data chunks, with 500–4000 samples per read at time of basecalling across different system configurations: AS using the fast model with disabled live basecalling (case 1, left), AS using the high accuracy (hac) model with disabled live basecalling (case 2, middle), and AS with the hac model running concurrent with hac live basecalling (case 3, right). OS indicates the Operating System used.

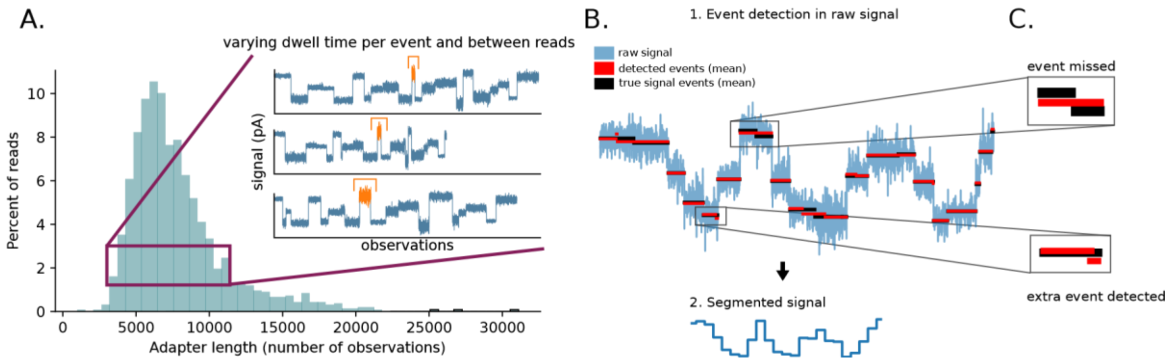

Supplementary Figure 35. Intrinsic variation in dwell time poses a source of noise. (A) Varying dwell time per event and between reads results in varying length adapter signals. (B) Segmentation of the raw signal into events, with each segment represented by its average signal, reduces signal noise caused by differences in dwell time and within-event measurement noise (C) Signal noise causes imperfect segmentation in which some events are missed while other spurious events are detected.

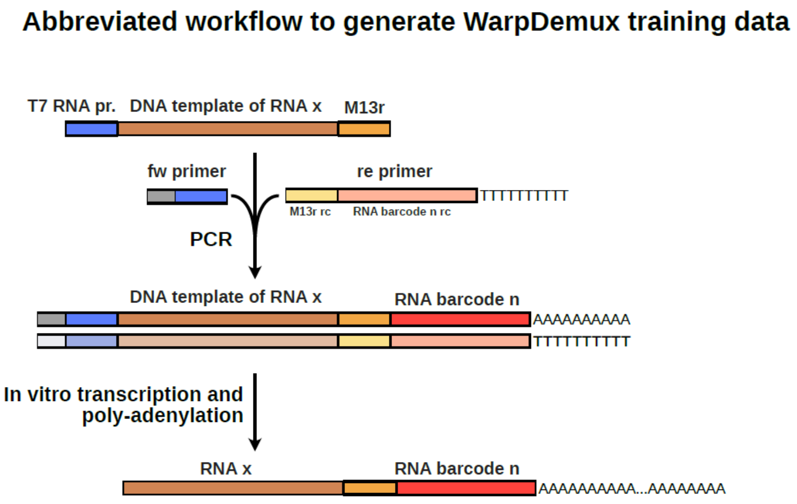

Supplementary Figure 36. Abbreviated workflow of experimental protocol to generate WarpDemuX training data.

Supplementary Method 1. Designing WarpDemuX barcodes

Experimental design

The RTA adapter consists of two partially complementary oligos. To ensure compatibility with the sequencing chemistry we designed the adapter oligos similarly to the tested design described in Smith et al.[1], with the exception of allowing variation of GC content. In detail, the two last nucleotides of the 5' end of the forward RTA adapter (GG) were kept constant to reduce potential ligation biases. In addition, all adapters retained the 3' adapter sequence that is required for the RMX motor protein to ligate onto. The reverse adapter was designed as reverse complement of the forward adapter, with the exception of a 10 nucleotide dT 3' overhang to hybridize to the RNA poly(A) tail, as well as a Y anchor sequence 5' of the reverse complement RTA barcode. All oligos were ordered from IDT with standard desalting purification, and with forward adapters containing a 5' phosphorylation. The following scheme exemplifies the design:

***In silico* design**

Barcode optimization addresses the need for a set of distinguishable signals that can be easily detected and separated. For barcodes of length *L*, an exhaustive search across all possible sets of *k* barcodes has complexity $\binom{4^L}{k}$, making such a search computationally infeasible: $\binom{6.9\times10^{10}}{12} \approx 2.3 \times 10^{121}$ for $L = 18,\ k = 12$.
Instead, we constructed a set of wave-like target signal patterns since we expect such patterns to improve segmentation, as well as distinction between barcodes and thus make for good barcodes in the context of our classification method.

Target patterns derivation

Across target patterns, we combined wave-like elements with diverse frequencies and amplitudes. Additionally, we maintained consistent minimal and maximal values across all target patterns to mitigate potential normalization biases during signal processing.

The customizable section of the RTA that serves as barcode spans 18 nucleotides. Given the sequencing k-mer size of 6, however, the preceding five constant nucleotides and the following constant two, as well as the first bases of the subsequent polyA tail also influence its signal. As a result, we expect 20 DNA and 3 DNA-polyA signal events. As there is no ONT k-mer model for mixed DNA-polyA k-mers, we assumed the signal of these k-mers to be an interpolation between the value of the last DNA k-mer and the expected signal level of a pure polA segment (∼ 110 pA). When constructing the target patterns, we substituted the interpolation k-mers with a singular polyA segment observation, thus yielding patterns that span 21 observations.

Candidate set construction

We searched for barcode sequences whose putative signal, constructed using the ONT k-mer models (github.com/nanoporetech/kmer_models), would closely match these target patterns. These models are designed for DNA sequenced in the 5' to 3' direction. In dRNA-seq, however, the RTA is sequenced in 3' to 5' direction. To increase the likelihood that our modeled barcode signals reflect the sequencing data, we limited our barcode design to the subset of k-mers for which the 5'-3' to 3'-5' directional reversal likely has little effect. Specifically, we selected k-mers with minimal variation (less than 10 pA) in expected signal mean when compared to their mirrored counterparts. For example, if the expected signal level difference between the k-mer AAACCC and the k-mer CCCAAA is < 10 pA, then the k-mer AAACCC is selected.

Subsequently, we discretized the expected signal values for the selected k-mers (1524 in total) and their mirrored counterparts respectively into five equally-sized, rank-based buckets. Only those k-mers whose original and mirrored versions both fell within the lowest, middle, or highest rank buckets were further considered, leaving us with 531 k-mers. This discretization and selection strategy enhance the probability of identifying wave-like patterns—marked by alternating low and high values—when assembling the k-mers into barcodes. Additionally, this approach strengthens the stringency of our heuristic for ensuring robustness against directional reversal.

From the filtered set of k-mers, we generated all conceivable combinations of five k-mers, adhering to a stride of three. This means that the final three bases of one k-mer overlap with the initial three bases of the following k-mer. This approach yielded roughly 3.4×10^6 candidate barcode sequences, each 18 bases long.

For each, we constructed the full adapter sequence by pre- and appending the flanking regions of adapter. Using the ONT k-mer models we constructed the putative adapter signal and appended it with a singular observation of the expected polyA tail signal. The full adapter signal was normalized to zero mean and unit variance. We then selected the last 21 observations of the idealized adapter signal, corresponding to the variable barcode part and calculated the distance to each target pattern.

Candidate selection

To select the set of $k = 12$ barcodes we selected the top 50 best-scoring (lowest distance) candidate barcodes per target pattern. For the $k = 12$ target patterns highlighted in Fig. 2A, this yielded a total of 600 unique barcodes, for which we computed all 600^2 pairwise DTWDs.

Lastly, within the matrix of 600^2 pairwise DTWDs we greedily approximate the Maximal Diversity Problem (MDP) to propose 12 candidate barcodes that we expect to be accurately identifiable and distinguishable. The greedy heuristic used to approximate the MDP was implemented as in Algorithm 1.

Algorithm 1: Greedy heuristic for solving the Maximum Diversity Problem.

```
input  : Distance matrix D ∈ ℝ_{>0}^{n×n}, Number of elements to select m
output: Indices of the selected elements
n ← number of rows in D;
first ← max_i(∑_j D_ij); // Select the element with the maximum sum of distances
selected ← [first];
while length of selected < m do
    maxMinDist ← −1;
    nextIndex ← −1;
    for i ← 0 to n − 1 do
        if i ∉ selected then
            minDist2Selected ← min{D_ij : j ∈ selected};
            if minDist2Selected > maxMinDist then
                maxMinDist ← minDist2Selected;
                nextIndex ← i;
            end
        end
    end
    Append nextIndex to selected;
end
return selected;
```

Supplementary Method 2. WarpDemuX signal processing

To account for the inherent variation in dwell time (Supplementary Fig. 35A), the raw signal is segmented (Supplementary Fig. 35B) by identifying the most significant shifts in current level based on the running difference between neighboring windows of raw signal. The pseudo code is described in Algorithm 2. Its parameters were tuned to reflect the translocation speed of the RTA, with a minimal segment length of 15 observations a sliding window width of 30 observations. Considering the known length of the adapter sequence, the signal is expected to contain 98 events. However, because segmentation is likely to be imperfect (Supplementary Fig. 35C), with missed events posing a greater challenge than spurious events, we intentionally oversegment the signal by approximately 10% to detect 110 events. Each segmented event is then represented by its average signal. Post-segmentation, segment means are normalized to zero mean and unit variance. Only the variable barcode component of the segmented signal, expected to be represented within the last 25 segments, based on the known barcode sequence length and location in the adapter, is used for classification.

Segmentation ensures that all inputs to the classification procedure are of uniform length. Due to the signal normalization, pairwise DTWDs are of similar magnitude across barcodes, negating the need for further scaling or normalization of the pairwise distance matrix.

Algorithm 2: Greedy algorithm for raw signal segmentation based on the most significant shifts in signal intensity.

input : Raw signal X of length N
$l \leftarrow 6$; `// Minimal segment length`
$k \leftarrow 12$; `// Width of sliding window`
$n \leftarrow 110$; `// Number of segmentation points to identify`
$\mathcal{W} \leftarrow \emptyset$; `// Empty list`
$\mathcal{T} \leftarrow \emptyset$;
$\mathcal{C} \leftarrow [0, N]$;
$\mathcal{S} \leftarrow \emptyset$;
for $i \leftarrow 1$ **to** $N - k$ **do**
  $w_i \leftarrow X[i : i + k]$;
  Append w_i to $\mathcal{W}$;
end
for $i \leftarrow 1$ **to** $N - 2k$ **do**
  $\bar{w}_i \leftarrow$ mean of w_i;
  $\sigma_i^2 \leftarrow$ variance of w_i;
  $\bar{w}_{i+1} \leftarrow$ mean of w_{i+1};
  $\sigma_{i+1}^2 \leftarrow$ variance of w_{i+1};
  $t_{i,i+1} \leftarrow \frac{|\bar{w}_{i+1} - \bar{w}_i|}{\sqrt{\sigma_i^2 + \sigma_{i+1}^2}}$;
  Append $t_{i,i+1}$ to $\mathcal{T}$;
end
while *size of* $\mathcal{C} < n$ **do**
  Find index i of max absolute value in T;
  Append $i + k$ to $\mathcal{C}$;
  Exclude values within range l of i from T;
end
Sort values in $\mathcal{C}$;
for *each consecutive pair* (c_1, c_2) *in* $\mathcal{C}$ **do**
  $s \leftarrow$ mean of $X[c_1 : c_2]$;
  Append s to $\mathcal{S}$;
end
return $\mathcal{S}$;

Supplementary Method 3. WarpDemuX parameterization

SVM

We use an C-Support Vector Classification SVM implementation based on libsvm[2], with a one-vs-one scheme for multi-class support, as implemented in Scikit-Learn[3] (scikit-learn 1.3.1).
With C, the regularization parameter, set to 10.0, kernel = ‘precomputed’ and default settings otherwise.

DTWD

To compute the Dynamic Time Warping Distance (DTWD) between two barcode fingerprints, we use the Python Package dtaidistance 2.3.10[4], with a window size of 15 (upper bound to allowed shift from the diagonal) and an additive warping penalty of 0.1 per compression or expansion.

Supplementary Method 4. Generating *in vitro* transcribed barcoded RNA

RNA barcoding strategy

In order to train and benchmark RTA-based barcoding strategies we generated *in vitro* transcribed RNAs of different templates containing varying RNA-encoded barcodes. This allows us to generate high accuracy ground truth labels for different RTA signals. To ensure that no signal spillover from RNA barcode into RTA signal was occuring we decided to enzymatically poly(A) tail these RNAs post *in vitro* transcription (although they already contain a 10 nt poly(A) tail that can be used for direct ligation of RTA adapters). Supplementary Fig. 36 provides a visual summary of the protocol. Further, we also decided to randomize RNA-barcode to RTA assignments to eliminate the possibility of inadvertently training on RNA signal in the rare possibility of RTA adapter signal failure Supplementary Table 1.

RNA barcode design

The RNA barcode sequences were designed with high RNA sequencing error rates in mind. Specifically, a large pool of 8 nt long barcode candidates was generated by random sampling, with some additional candidates supplemented by BARCOSEL[5], and a set of 18 barcodes that maximized inter-barcode Levenshtein edit distances was chosen. To balance purine and pyrimidine content in each barcode and double the number of variable nucleotides in the final barcodes a self-complementary stem-loop (i.e. [barcode]-GAGUA-[revcomp_barcode]) was generated for each barcode. To introduce these RNA barcodes into DNA templates reverse primers containing a 10 nt long T stretch at their 5' end, followed by the reverse complement of the barcode stem-loop, and finally the reverse complement of the M13-reverse sequence were ordered with standard desalting purity from IDT (Supplementary Table 25).

PCR to generate barcoded DNA templates

PCR was performed on templates of the Vibrio cholerae glycine riboswitch, hc16 ligase, bacterial RNase P type A, tetrahymena riboswitch and Hepatitis C Virus IRES cloned into pJet (Thermo Fisher) flanked by a T7 RNA promoter sequence and an M13r sequence at 5' and 3' ends respectively as described in Bohn *et al.*[6] using Primestar GXL (Takara) according to the manufacturer's instructions. Reaction volume was 20 ul, annealing temp. 50 °C, extension time 30 seconds at 35 cycles with forward primers described in Supplementary Table 26 and reverse primers as described in Supplementary Table 25.

***In vitro* transcription**

PCR products were quality controlled via agarose gel and purified by addition of 18 µL H2O and 38 µL SPRI beads (Omega Biotek NGS). After hybridization for 5 min under light agitation (300 rpm on hula mixer) beads were pelleted using a DynaMag-96 Side Magnet (Invitrogen). Supernatant was removed, beads were washed twice with 100 µL 80% EtOH and eluted in 20 µL H2O. DNA concentration was measured with Nanodrop and 250 fmol of DNA was then used in a 10 µL *in vitro* transcription reaction: 5 mM NTPs, 5 U/µL T7 RNA polymerase (homemade), 1 µL YIPP (NEB), 0.1 µL RNasin (Molox) in 40 mM Tris pH 7.5, 18 mM $MgCl_2$, 10 mM DTT, 1 mM spermidine, 5 mM NTPs, 40 U RNasin (Molox) and homemade T7 RNA polymerase. After *in vitro* transcription for 2 h at 37 °C the DNA template was digested by addition of 10 µL of DNase mix containing 1 µL 10x DNase I buffer and 0.2 µL of DNase I (NEB) followed by a 15 min incubation at 37 °C. The RNA was then purified by addition of 1.4 volumes of SPRI beads and two washes with 80% EtOH. Purified RNA was then quality controlled with a 1.5% agarose gel in 1x TAE and concentration was quantified via Nanodrop.

Poly(A) tailing

For poly(A) tailing 1 ug of IVT RNA was incubated with 0.5 µL E. coli poly(A) Polymerase (NEB), 1 µL 10 mM ATP in 1x poly(A) polymerase reaction buffer in a 10 µL reaction for 30 min at 37 °C, followed by purification with 1.4 volumes of SPRI beads and two washes in 190 µL 80% EtOH before elution in 20 µL RNase-free H2O.

Supplementary Method 5. Direct RNA sequencing library preparation

Annealing of RTA adapters

The forward and reverse strands of the RTA oligos were annealed following a procedure similar to that described in Smith et al.[1]. Specifically, oligos were diluted to 10 µM each in 30 mM HEPES-KOH (pH 7.5), 100 mM K-Acetate, heated to 95 °C for 1 min and cooled to 25 °C at a rate of 0.5 °C/min. The annealed RTA adapters were then diluted to 1.4 µM with RNase-free H2O and stored at -20 °C until use.

RTA adapter ligation

Volumes were scaled for direct RNA sequencing on a Flongle flow cell according to the protocols.io protocol of Krause[7]. In detail, for each sample, 100 ng of poly-adenylated *in vitro* transcribed RNA in 4.4 µL, was transferred into a PCR tube, followed by addition of 1 µL previously annealed custom RTA adapter, 1.6 µL 5x NEBNext Quick Ligation Buffer (NEB) and 1 µL T4 DNA Ligase high concentration (NEB). The reaction was mixed well by pipetting and incubated at room temperature for 20 min. To stop the ligation 2 µL of 0.5 M EDTA was added to each reaction, samples were pooled, purified with 0.8 volumes of SPRI beads washed twice with 80 % EtOH, and eluted in 20 µL RNase-free H2O.

Reverse Transcription

To perform the reverse transcription a master mix containing 1.9 µL H2O, 15.7 µL 3x MarathonRT buffer (150 mM Tris-HCl pH 8.3, 600 mM KCl, 60 % (v/v) glycerol), 2.35 µL 100 mM DTT, 0.2 µL RNasin (Molox), 1.9 µL 50 mM MgCl2, 3 µL of 10 mM dNTPs and 2 µL MarathonRT (produced in house [6]) was prepared and added to the 20 µL RNA-RTA ligation. After incubation at 42 °C for 60 min the reaction was stopped by addition of 2 µL Proteinase K (NEB), followed by another incubation at 42 °C for 15 min. The reaction was purified with 1 volume SPRI beads, washed thrice with 190 µL 80 % EtOH, and eluted in 30 µL RNase-free water (volume was increased to adjust for higher total input amount due to multiplexing).

Motor protein ligation

To proceed with Nanopore sequencing we next ligated on the motor protein. For this we transferred 10 µL of purified reverse-transcribed RNA-RTA library into a 1.5 ml DNA LoBind tube (Eppendorf), followed by addition of 2.5 µL H2O, 4 µL 5x NEBNext Quick Ligation Buffer (NEB), 2 µL RMX motor protein adapter (ONT SQK-RNA002), and 1.5 µL T4 DNA Ligase high conc. (NEB). The reaction was mixed by pipetting and incubated for 20 min at RT, followed by purification with 20 µL SPRI beads, washed twice with RNA wash buffer (WSB, ONT SQK-RNA002). The library was then eluted in 9 µL elution buffer (EB, ONT SQK-RNA002) and 8 µL was transferred into a new 1.5 ml DNA LoBind tube. The library was then loaded onto a Flongle FLO-FLG001 flow cell for sequencing.

Supplementary Method 6. Data acquisition and basecalling software configuration

Data acquisition was performed using MinKNOW version 22.12.7. Basecalling was performed using different configurations depending on the sequencing chemistry used:

RNA002 chemistry

Basecalling was performed using dorado version 0.3.4 with the following configuration:

- Protocol: MIN106_RNA:FLO-FLG001:SQK-RNA002
- Model: rna002_70bps_hac@v3

RNA004 chemistry

For RNA004 chemistry, dorado version 0.7.3 was used with:

- Model: rna004_130bps_sup@v5.0.0

Supplementary Method 7. Live WarpDemuX system configuration

The live demultiplexing system was deployed on a standard workstation with the following specifications:

- CPU: 12th Gen Intel(R) Core(TM) i7–12700, 2100 Mhz, 12 Cores
- RAM: 32 GB
- Operating System: Windows 10 Pro

Supplementary Note 1. Training set selection, mislabeled instances and Noise Class

We calculated the pairwise Dynamic Time Warping Distances (DTWDs) for all barcode fingerprint instances outside of validation or test sets. First, we identified potentially mislabeled instances based on the robust Zscore of their median within-class distance. Reads with an absolute value of at least the 98th percentile were excluded from the data set.

Upon inspection of the excluded data, we noticed that a substantial part of these reads were the result of a sequencing artifact known as a missed read split. The two common types were: (1) a concatenation of two complete reads, each consisting of an adapter, polyA and RNA signal (Supplementary Fig. 1a), and (2) a concatenation of an 'adapter-only' read and a complete read, wherein the first consists of an adapter and subsequent polyA tail signal, but misses the RNA signal (Supplementary Fig. 1b).

In both cases, the adapter signal of the first read in the read pair is detected, processed and classified. However, the ground truth label for this read, inferred based on a combination of the basecalled RNA transcript and RNA barcode, may belong to the second read of the concatenated pair, rather than the one processed. After filtering out mislabeled instances, we calculated the robust Z-scores of the median inter-class distances to identify noisy signals. For this, we used the minimal absolute value score across classes per instance. Reads with a minimal absolute value score above the 99th percentile value were labeled as noise. From these, we randomly sampled 400 instances as training data for the noise class.

Upon inspection of the noise class data, we observed stalls to be the main type of signal irregularity, potentially leading to mis-segmentation of the adapter itself (Supplementary Fig. 2a). In addition, we observed stalls in the beginning of the adapter to cause mis-identification of the DNA/RNA boundary (Supplementary Fig. 2b).

For the remaining reads, we approximate the solution of the Maximal- and Minimal Diversity Problem, stratified across barcode classes (Algorithm 3 and 4), to select 2×80 instances per barcode that respectively maximized and minimized their mutual distance. The training set was completed with 240 randomly sampled instances per barcode.

Algorithm 3: Greedy heuristic for solving the Maximum Diversity Problem, stratified over classes.

```
input  : Distance matrix D ∈ R_{>0}^{n×n}, Number of elements to select m
output: Indices of the selected elements
n ← number of rows in D;
C ← Class labels of elements in D (rows);
selected ← []; // Empty list
for c ∈ unique(C) do
    first ← max_i(∑_j D_ij) : j ∈ selected AND i ∈ {C_i == c}; // Select the element with the
        maximum sum of distances per class
    Append first to selected;
end
while length of selected < m do
    for c ∈ unique(C) do
        maxMinDist ← −1;
        nextIndex ← −1;
        for i ← 0 to n − 1 do
            if i ∉ selected AND C_i == c then
                minDist2Selected ← min{D_ij : j ∈ selected};
                if minDist2Selected > maxMinDist then
                    maxMinDist ← minDist2Selected;
                    nextIndex ← i;
                end
            end
        end
        Append nextIndex to selected;
    end
end
return selected;
```

Algorithm 4: Greedy heuristic for solving the Minimum Diversity Problem, stratified over classes.

```
input  : Distance matrix D ∈ R_{≥0}^{n×n}, Number of elements to select m
output: Indices of the selected elements
n ← number of rows in D;
C ← Class labels of elements in D (rows);
selected ← []; // Empty list
for c ∈ unique(C) do
    first ← min_i(∑_j D_ij) : j ∈ selected AND i ∈ {C_i == c}; // Select the element with the
        minimum sum of distances per class
    Append first to selected;
end
while length of selected < m do
    for c ∈ unique(C) do
        minMaxDist ← 1000;
        nextIndex ← −1;
        for i ← 0 to n − 1 do
            if i ∉ selected AND C_i == c then
                maxDist2Selected ← max{D_ij : j ∈ selected};
                if maxDist2Selected < minMaxDist then
                    minMaxDist ← maxDist2Selected;
                    nextIndex ← i;
                end
            end
        end
        Append nextIndex to selected;
    end
end
return selected;
```

Supplementary Note 2. Best barcode sets and recommended confidence cutoffs (RNA002)

Based on a 10-fold cross-validated benchmark, we selected the best performing subsets of barcodes for experimental settings requiring less than 12 barcodes (Supplementary Table 9). The trained WarpDemuX models for these barcode subsets are available in the code repository.

To facilitate a fair comparison between models with a different number of barcodes, as well as to improve the ease-of-use for the user, we introduce two modes of operation per model: a High Accuracy mode (HA) and a High Recovery mode (HR), with respectively tuned confidence cutoffs based on the performance on the validation set.
For the mode HA, the confidence cutoff is set to the value for a 90% yield on the validation set. For mode HR, the confidence cutoff is set to the value for a 95% yield on the validation set. The respective performances per dataset are reported in Supplementary Table 12.

Supplementary Note 3. Short poly(A) tail length

Notably, WarpDemuX signal preprocessing hinges on accurately detecting the (start of the) poly(A) tail in the raw signal, which becomes increasingly challenging as the poly(A) tail length decreases. Despite this, the tool also achieved high demultiplexing performance on RNA with short polyA tails (as in dataset 6). This indicates that WarpDemuX can also be employed in experimental settings where enzymatic poly-adenylation is not feasible, such as in the studies were the length of the poly(A) tail is of interest[8-11].

Supplementary Note 4. Analysis of SARS-CoV-2 sequencing run

Vero cells were infected with recombinant SARS-CoV-2 wildtype or SARS-CoV-2-TurboGFP, replacing ORF8.
This results in distinct subgenomic RNAs being expressed by either variant (Supplementary Fig. 8). Cellular RNA and supernatant RNA was extracted using phenol/chlorofom and barcoded with WDX barcodes as described in Supplementary Table 27.

Run statistics

After sequencing, reads were filtered for a mean Phred quality score of 10 (Supplementary Fig. 9a). All reads were then passed through the WarpDemuX pipeline. Notably, we observed a relation between the basecall-based read Phred qscore and the WDX confidence score distribution (Supplementary Fig. 9b-e), indicating that there is a common underlying source of noise that can affect both basecalling and WDX demultiplexing confidence[12].

Read to species assignments

Next, reads were aligned with minimap2 to the Vero reference genome (Genbank: GCA_000409795.2), SARS-CoV-2 genome (NCBI RefSeq: LC528233) or TurboGFP sequence (NCBI RefSeq: GU452685.1 306 to 999):

```
minimap2 -ax splice -u f -k14 {reference_index} {fastq_file} > {bamfile}
```

Uniquely mapped reads to each reference were extracted with samtools:

```
samtools view -F 0x100 {bamfile} | awk '{{print $1, $3}}' > {outfile}
```

When a multi-mapping read was detected it was assigned to ref_A–ref_B (for example reads that aligned to both SARS-CoV-2 and TurboGFP were assigned to SARS2-TurboGFP). Those aligning to multiple Vero reference contigs were assigned Vero. Already at a WarpDemuX confidence threshold of 0 the vast majority of reads classified as a given barcode were of the expected species origin. Only for the two highly underrepresented barcodes 6 and 12 (supernatant samples) some SARS2-GFP reads were detected, which are likely from a low level of spillover from other samples that becomes substantial with low number of true reads (Supplementary Fig. 10).

WarpDemuX confidence threshold choice

The WarpDemuX confidence score represents the confidence of the classifier to correctly classify a given adapter signal. The confidence threshold below which reads are assigned as unclassified can be chosen depending on the experimental setup and requirements and results in a tradeoff between yield and accuracy (Supplementary Table 5). In this case, for heavily unbalanced input containing two rare (supernatant) samples we determined that a highly stringent cutoff must be chosen to ensure high specificity even for these rare samples.

To further illustrate the importance of focusing on high-confidence classifications to ensure reliable biological interpretation when working with highly-imbalanced datasets, consider the following example: Consider a dataset with three samples of varying abundances: sample A and C (high abundance) and sample B (very low abundance). Even if the likelihood of reads from sample A being misclassified is low, if an inadequate confidence threshold is chosen, the impact of these misclassifications varies depending on the target sample's abundance. While the absolute number of misclassified reads from sample A to samples B and C may be identical, their relative impact differs substantially. For the low-abundance sample B, these misclassified reads constitute a large proportion of its total signal, potentially masking the true biological signal. In contrast, for the highabundance sample C, the same number of misclassified reads represents only a small fraction of its total signal, having minimal impact on downstream analysis. This demonstrates that the impact of misclassification must be considered in the context of the target sample's abundance. By enforcing an adequately high confidence threshold, the spillover can be minimized, ensuring reliable biological interpretation across all abundance levels. Upon evaluating the confidence score distributions per sample (Supplementary Fig. 11) a confidence threshold of .99 was chosen to optimally balance demultiplexing accuracy with yield. While this resulted in approximately 32% unclassified reads across all samples (Supplementary Fig. 12a), the fraction of reads below a WarpDemuX confidence score of 0.99 was substantially higher for the two rare samples bc6 and 12 (80% and 64% respectively) (Supplementary Fig. 12b), consistent with higher relative levels of spillover expected. Indeed, with this stringent threshold almost no Vero reads were now detected in the supernatant samples, as well as no GFP reads remaining in the sample with bc06 (Supplementary Fig. 12c). When low-confidence predictions were included, this clear sample separation deteriorated, confirming our hypothesis about the importance of stringent thresholds.

When evaluating the read lengths after WarpDemuX demultiplexing through a virtual gel, bands presumably corresponding to SARS-CoV-2 isoforms became immediately apparent (Supplementary Fig. 13). For samples with the GFP variant, the bands above 1.5 kb were shifted approximately 500 bp upwards, consistent with the expected increased length of sgRNAs due to the GFP insert (Supplementary Fig. 8).

SARS-CoV-2 read transcript assignments

To confirm that these reads with distinct lengths were full length SARS-CoV-2 subgenomic RNAs, their TRS-L sequence was searched for using cutadapt v4.8 was used with the following settings:

```
SARS2_5UTR="ATTAAAGGTTTATACCTTCCCAGGTAACAAACCAACCAACTTTCGATCTCTTGTAGATCTGTTCTCTA"
cutadapt -g $SARS2_5UTR -e 0.25 --overlap 10 --action=none --untrimmed-output
```

Reads where the TRS-L sequence was found were marked as full-length, while those without TRS-L sequence were labeled as incomplete. When evaluating read lengths of the so classified reads, indeed reads where the TRSL sequence was present showed distinct lengths (Supplementary Fig. 14), which was subsequently used for isoform assignment by length (Supplementary Fig. 8). If the reads were too short to be distinguished between the GFP and non-GFP variant of the virus, they were assigned as ambiguous.

After assignment of SARS-CoV-2 isoforms, the relative abundance of full length and incomplete SARS-CoV-2 reads per sample could be estimated, revealing a constant ratio of full length to incomplete reads for the cellular samples throughout infection, while the two supernatant samples showed distinct reduction of full length reads (Supplementary Fig. 15), consistent with the assumption that subgenomic RNA is selected against during packaging.

Vero read transcript assignments

To differentiate RNA types within Vero-aligned reads, we performed read classification with Isoquant v3.3.1. We used transcript annotations contained in the Genbank entryy GCA_000409795.2, as these resulted in higher number of classified reads in comparison the more recent reference GCA_015252025.1. The specific settings used were:

```
isoquant.py --data_type nanopore --stranded forward --complete_genedb --reference {fasta}
        --genedb {gtf} --bam {' '.join(bam_files)} --labels {' '.join(samples)} -o {outdir}
```

Finally, descriptive gene and transcript type annotations were acquired through Ensembl BioMart and merged into the isoquant read classification. This revealed that the vast majority of Vero-aligning reads were from protein-coding genes, as expected from sequencing poly-adenylated RNA. A minor fraction of reads were assigned to mitochondrial rRNA however, possibly due to mitochondrial poly-adenylation of non-coding RNA (see Supplementary Fig. 16a).

Poly (A) tail length estimation

To estimate the poly(A) tail lengths of SARS-CoV-2 RNA in cells and virion as well as the effect of SARS-CoV-2 infection on cellular mRNA poly(A) tail length we performed tail length estimation with tailfindr[10]. Specifically, we converted the raw pod5 files into fast5 files before rebasecalling with guppy 6.3.4 with "–fast5-out" option. We then ran tailfindr version 1.4 on the generated fast5 files and incorporated the per read poly(A) tail estimations as provided in the csv file into our analysis. By leveraging annotation information of Isoquant-assigned transcripts we were also able to study the polyadenylation landscape of Vero-derived transcripts. We detect distinct poly(A) tail length distributions between nuclear (50–150 nt) and mitochondrial (10–50 nt) transcripts (Supplementary Fig. 17a), as well as distinct mean length differences between SARS2- and Vero-derived transcripts (Supplementary Fig. 17b). Intriguingly, as highlighted in the results, a distinct bimodal distribution of poly(A) tail lengths was apparent for SARS2 transcripts at 72 hours post infection (hpi), with the smaller tail length population being less abundant at earlier time points. A bimodal Gaussian Fit revealed the modes of these two distributions to be 52 nt and 29 nt respectively (Supplementary Fig. 17c), with components $N_1(29.44,5.89)$ and $N_2(51.80,20.17)$ and mixing coefficients 0.148 and 0.842, respectively. Leveraging our longitudinal samples, we examined the dynamics of the poly(A) tail length in SARS2 cellular transcripts. We then applied the expectation-maximization (EM) algorithm to estimate the mixing coefficients for the 24 and 48 hpi data, while keeping the distribution components fixed. This yielded coefficients of 0.001 and 0.999 for 24 hpi, and 0.272 and 0.973 for 48 hpi.

Supplementary Note 5. Appendix-software adaptations

Live barcode balancing in direct RNA sequencing has the prospect to substantially increase yield for multiplexed sequencing runs, as well as to increase pore half-life when some, but not all samples that are sequenced have a high likelihood of blocking pores. It therefore can reduce labour and resource costs, making dRNA-seq a more viable solution to prevalent questions related to epitranscriptomics.

Of note is that adaptive barcode balancing will not alter the relative read abundance of barcodes, but it will result in higher turnover of reads, thus leading to a decreased of sequenced bases for rejected and concurrent increase of bases for accepted barcodes.

To facilitate live barcode balancing, multiple steps need to be performed with high accuracy, yield and speed: (1.) streaming data quickly from the sequencing device, (2.) identifying and extracting adapter signals from the start of a read until the transition to polyA tail/RNA, (3.) rapid processing and classification of the adapter signal, and lastly, (4.) decision on rejection and if so, sending the command to the sequencer (Supplementary Fig. 18).

For dRNA adaptive barcode sampling to result in substantial enrichment when compared to a standard sequencing run, the time from the read starting to enter the pore to the decision should be as short as possible. To estimate theoretical limits of enrichment, we can Additionally, rejection of RNA reads should not lead to a substantial increase in pore loss.

One possible source of pore loss can be the RNA refolding at the trans side of the pore. To reduce the likelihood of this, we aim to reject reads while the poly(A) tail is still in the pore. To estimate the required turn-around time for this goal for RNA002 we calculate the following: DNA adapter translocation takes approximately 2 seconds, followed by translocation of RNA nucleotides at 70 bases per second. At a poly(A) tail length of 30 nucleotides, this means optimally we want to achieve a time of up to 430 ms from poly(A) start to decision. The adaptive sampling process is accessible through the read_until_api, which acts as an interface between the gRPC interface in MinKNOW and custom python code. Matt Loose's team leveraged this API to develop the first iteration of DNA adaptive sampling[13], adding a critical component, an accumulating cache, to the read_until_api. This cache is critical for us because we require the near whole adapter signal at once (up until the poly(A) signal starts) to be able to segment and classify properly, and this signal is highly likely to stretch multiple individual chunks.

Reducing the chunk size
Chunks are blocks of signal that are received through the read_until_api. The size of chunks is determined in the sequencing protocol toml file, specifically by the break_reads_second parameter. By default chunks consist of 1 second of signal. However, as we attempt to achieve low latency, reducing the chunk size was a critical step. Indeed, reducing the break_read_seconds parameter to 0.1 second resulted in receiving chunks of size 300 samples, enabling rejection during or shortly after the poly(A) tail has passed the pore.

However, this change also resulted in nearly no reads being generated by MinKNOW. Investigating this issue further, we identified the read_classification parameters as well as the channel_states as the two key steps that required tuning. The reason is as follows: first, new reads are detected by an open pore event (which is not bound by the chunk size). Then, each chunk gets classified according to the boundaries specified in the read_classification parameters (duration means duration since the read started). The channel state is then determined by regex matching of patterns of chunk classifications, those are defined in the channel_states.toml file.

As we decrease the chunk size, statistics such as median and range (90%-10% percentiles) become less consistent, and we identified these as the parameters that required the most tuning. Critically, the range was previously used to detect stalled reads, as it was unlikely (apart from the poly(A) tail) that the signal stayed within a small range for 1 second. In contrast, it may happen relatively frequently that the signal may stay constant in a 0.1 second window—thus we decided to decrease the minimum range required. Upon replaying the simulation, these changes not only recovered the output of the initial sequencing run, but increased it by approximately 25% (data not shown). However, this change in isolation may lead to stalls not being detected, thus severely affecting overall flow cell yield by not recovering blocked pores. To address this problem we created a new read_classification state stalled. Since brief stalls are expected to occur within a read, we tolerate up to 3 'stalled' chunks in between two 'strand' chunks before the channel_state is changed from strand to stalled. Only when at least 10 chunks are consecutively classified as 'stalled' we initiate the unblocking event by changing the channel_state to 'blocked'. With these necessary adjustments, our custom protocol's performance became comparable to the default protocol in terms of read count, length, and accuracy (Supplementary Fig. 19).

While these changes were successful for our sequencing experiment, we want to state the caveat that the interplay of chunk size with channel classifications, and thus reject decisions, is highly complex, and likely requires multiple iterations of empirical tuning to be functional. In particular, in our selected protocol, we saw an increase in overall rejection events (Supplementary Fig. 20a). Some of this increase may be attributed to the shorter chunk size enabling faster detection and repeated triggering of unblock commands. Indeed, the majority of unblock commands issued by the default protocol are also recapitulated by the 100 ms chunk size protocol (Supplementary Fig. 20b), and the majority of 100 ms chunk size unblock commands also fell within 5 seconds of an unblock command issued by the default protocol (Supplementary Fig. 20c). Nevertheless, a substantial part of unblock commands of the 100 ms chunk size protocol occurred during timeframes where the default protocol had classified the signal as a read (adapter/strand) (Supplementary Fig. 20d). By analyzing at which position in the read these reject commands were issued (Supplementary Fig. 20e), we could identify two distinct failure modes of our current protocol: an early rejection event (within 0–10% of read duration) issued in the adapter sequence, often briefly after the signal briefly spiked over 100 pA, and a mid-rejection event (10–60% of read duration) occurring within the poly(A) signal (Supplementary Fig. 20f). These failure modes reiterate the complexity involved in altering the sequencing protocol, and reveal areas of future optimization.

Read until API changes
Having achieved a 100 ms chunk size, we next focused on the read until API. The accumulating cache that appends chunk raw signal (instead of overwriting it) is critical for live barcode classification, especially with the decreased the chunk sizes. However, we noticed that for some reads, the start of the signal did not match the start of the new read, and this may result in incorrect poly(A) detection, segmentation or classification. We identified the source of this to stem from a filter on incoming chunks according to their classification implemented in the accumulating cache, and only accepting adapter- or strand-classified chunks. While this is reasonable for basecaller-based adaptive sampling, it can lead to the mentioned data loss at the start of reads due to the possibility of other types of chunk classifications. As such, we have removed this filter during chunk accumulation, but instead added a filter in the get_read_chunks function that in our case will only return accumulated chunks that contain at least one adapter classification
(see our implementation at https://github.com/KleistLab/WarpDemuX). In addition, we added a max length to the accumulated raw data to ensure that raw signal of long reads or inactive channels does not grow infinitely.

Decision strategies
The last step after classifying raw signal of adapters is to decide on the fate of the read. The simplest approach would be rejection based on relative adapter occurrence. However, this has drawbacks with regards to unligated adapter sequences, which when evaluating raw data may be present in 50% of adapter events, and potentially at varying abundance between different samples.

Instead, we propose two alternative strategies, that both employ additional information present in pod5 files that are generated during the sequencing run. In both strategies we first classify adapters and store their read ids. We then search in the pod5 files as they are generated during the run to identify matching read ids, counting all matches for each barcode. As adapter-only reads are not written out to pod5 files this ensures that we only normalize on true RNA-containing reads. Intriguingly, the output of this analysis can subsequently be used to estimate the fraction of adapter-only reads in the library, helping to troubleshoot library preparation protocols. In the second strategy we perform the same lookup in pod5 files, but integrate the number of detected events by MinKNOW. These events correspond closely to the number of bases in the read (with approximately 100 additional events introduced by the adapter), so incorporating this information allows us to balance different samples not only by read count, but by base count.

Similarly, if samples are suspected to result in high levels of blockage, we can also identify barcodes that resulted in higher than expected rates of pore blockage by monitoring the end reason of reads in pod5 files, and flag those samples for rejection in order to extend pore lifetime.

Supplementary Note 6. Live barcode balancing

Latency of WarpDemuX adaptive-sampling

WarpDemuX's classification speed allowed the implementation of barcode-specific adaptive sampling for dRNA-seq. Our goal was to make rejection decisions while the poly(A) tail of RNA molecules is still within the pore such that (i) rejected reads do not end up in the sequencer output folder (fast5_pass/pod5_pass) and (ii) the risk of pore blockage upon rejection is reduced by preempting the formation of RNA structures on the trans side of the pore that may hinder ejection of the molecule back through the pore.

Indeed, when evaluating the rejected reads after completion of the adaptive-sampling run, most rejected reads were too short to be written out to pod5 files (Supplementary Fig. 21a), with rejection request successfully executed within 400 ms after detection of the polyA tail for nearly all reads (Supplementary Fig. 21b). Reads that were rejected but still written out to pod5 files exhibited shorter read lengths compared to retained reads (Supplementary Fig. 21c).

Efficiency of WarpDemuX adaptive-sampling

The efficiency of adaptive sampling for typically short mRNAs warrants careful examination, particularly regarding the impact of latency on potential enrichment. Our analysis focuses on the time spent sequencing rejected reads (the interval between molecule entry in the pore and registration of the adaptive-sampling decision), comparing this to the theoretical 'passive' (no adaptive sampling) sequencing duration.

We first investigated the distinct translocation speeds for the adapter and RNA segments of each read. Using this data, we modeled the passive sequencing time for molecules of varying lengths and compared these projections against observed sequencing durations prior to decision. This comparison revealed the potential time savings per rejected read—a direct measure of adaptive sampling efficiency and achievable enrichment.

Since rejected reads rarely appear in pod5 files (> 85% are too short), we used retained reads to estimate translocation speeds, avoiding bias from outliers or unusual translocation speeds in the few rejected reads that are written out. Analysis of our SARS-CoV-2 dataset uncovered several key findings. The adapter region exhibited slower translocation (55.7 nt/s) compared to RNA (70.8 nt/s), representing a 1.3-fold difference (Supplementary Fig. 22a). Breaking down the timeline from molecule entry to decision showed that adapter sequencing consumed most of the time (1.9s; 84.7%). WarpDemuX operations added little overhead, with latency accounting for 12.8% (median 284 ms) and the adaptive-sampling analysis requiring just 1.8% (median 41 ms) of total sequencing time (Supplementary Fig. 22b, Supplementary Fig. 23).

By sampling from observed translocation speed distributions, we estimated time savings across different template lengths. For molecules exceeding 300 nucleotides, over 90% of rejected reads would save more than half the sequencing time. This efficiency increased with length—templates of 500 and 1000 nucleotides showed minimum savings of 65% and 80% respectively for 90% of reads (Supplementary Fig. 22c).

The theoretical maximum enrichment for RTA-based adaptive sampling, with barcodes placed in the customizable section at the end of the adapter, would occur under zero latency and instantaneous processing. Under these ideal conditions, only adapter sequencing time would factor into the molecule-start-to-decision interval. Remarkably, WarpDemuX performance approaches this theoretical maximum for templates longer than 300 nucleotides, despite real-world latency and processing requirements (Supplementary Fig. 22d).

Balancing strategies

Relative barcode abundances can be balanced based on different statistics. We developed multiple balancing strategies, such as barcode balancing based on adapter counts, read counts, or number of bases (Supplementary Note 5). We also implemented barcode blacklisting, where certain barcodes are automatically rejected, independent of the barcode balance.

To evaluate the performance of the different methods, we applied each to 40 channels each on a MinION R9.4.1 flow cell, leaving 312 channels to sequence without adaptive-sampling for comparison. Balancing strategies resulted in a more even read count over barcodes, and the blacklist logic depleted targeted barcodes only, as expected (Supplementary Fig. 24). All balancing strategies resulted in enrichment of low abundance samples, both in terms of number of reads and number of bases sequenced (Supplementary Fig. 25, Supplementary Table 13).

Pore lifetime

Ultra-fast barcode classification with adaptive-sampling can lead to rejection frequencies above 50% (Fig. 4e), depending on the abundances of the multiplexed samples and balancing strategy. An unsuccessful rejection may result in pore blockage, reducing the lifetime of the pore and sequencing yield of the run. We aimed to minimize the number of pore blockages upon unsuccessful rejection by rejecting reads while they are still within the poly(A) tail. We analyzed the effect of different balancing strategies on pore lifetime, and found no substantial differences between pores with and without adaptive sampling, regardless of the strategy used (Supplementary Fig. 26).

Supplementary Note 7. Temporal perturbation analysis between DTWD and GASF

While the DTWD is resilient to temporal perturbations, the GASF feature transformation exhibits vulnerability to such perturbations (Supplementary Fig. 31). Consequently, stochastic fluctuations in dwell time introduce significant noise when employing a GASF-based approach. Although DeePlexiCon shows that a CNN can learn to discern between genuine correlations and noise in the signal-transformed image, the inherent complications of the GASF transformation intensify the classification task. This is manifested in the augmented training data requisite for the CNN and its diminished performance in comparison with WarpDemuX.

Supplementary Note 8. Cost-benefit analysis of multiplexed sequencing

We have sequenced our RNA004 dataset using a PromethION flow cell, demonstrating that WDX is flow cellindependent and applicable in production settings. While multiplex sequencing may not be viable for use cases requiring exceptionally deep coverage, the benefits of pooled sequencing of barcoded samples have been well-established for most applications and can reduce costs by an order of magnitude. Notably, ONT does not provide a Flongle flow cell for the new RNA004 chemistry, necessitating that each sample be sequenced on a MinION or PromethION flow cell, with multiple rounds of washing, which substantially increases hands-on time and cost per sample. Our multiplexing strategy reduces the cost per sample below that of single Flongle flow cells while increasing the output per sample, as PromethION flow cells yield approximately 50-fold more data (practical yield for RNA, PromethION: 50 Gb vs. Flongle: 1 Gb).

Below, we provide detailed calculations on costs and expected reductions in the context of maximal data yield expectations (Supplementary Table 20-23). Values were calculated using the following estimates:

- Practical yield of MinION flowcell, RNA: 10 Gb
- Practical yield of PromethION flowcell, RNA: 50 Gb
- Number of times a flowcell can be washed: 5 times
- Maximal number of samples to be multiplexed together on a single flowcell: 24 samples
- Yield of demultiplexing workflow: 85%
- Costs of Library Preparation Kit: e600 (6 runs per kit)
- Costs of Flowcell Wash Kit: e100 (6 runs per kit)
- Costs of Flowcell: e800
- Cost of Labor: e60.00/hour
- Hands-on time required: 30 minutes for washing flow cell, 1 hour for preparing library and 15 minutes for flowcell loading, per flowcell

Supplementary Note 9. Analysis of basecalling-based adaptive sampling latency

Introduction

The efficiency of adaptive sampling in nanopore sequencing depends critically on the time between data acquisition and rejection. In this context, latency—defined as the time delay between initiating an action and receiving a response—encompasses multiple components beyond mere processing time. These components include data transmission delays, queue waiting times, storage access delays, and various other system-level delays. Importantly, a system may achieve high processing speeds while still experiencing significant latency due to these cumulative delays.

While basecalling-based approaches offer high accuracy, their computational overhead may impact real-time performance. Basecalling-based approaches rely on the use of GPU-computing which, despite achieving high throughput through simultaneous processing of many inputs, incur additional latency from signal batching, data transfer between host and GPU device, and computational kernel initialization. This appendix presents an analysis of the inherent latency in basecalling-based adaptive sampling approaches, specifically examining performance with 0.1-second data chunks to match WarpDemuX specifications.

Methods
We measured basecalling latency by replaying a run that matched the settings used for WarpDemuX adaptive sampling (break_read_after_seconds=0.1, 500–4000 samples submitted to classification). In order to evaluate the computational overhead, we tested baescaller adaptive sampling latency in three configurations:

Case 1: Fast model configuration: Adaptive sampling using the fast model with disabled live basecalling. This represents the best case scenario for adaptive sampling

Case 2: High accuracy configuration: Adaptive sampling using the high accuracy (hac) model with disabled live basecalling. This represents increased accuracy adaptive sampling

Case 3: Concurrent processing configuration: Adaptive sampling with the high accuracy model running concurrent with live basecalling (hac model). This represents the setting that is most similar in accuracy to live WarpDemuX: During our live WarpDemuX experiments, RNA transcripts from accepted reads underwent live basecalling (hac model).

For each configuration, data was polled using read_until_client.get_read_chunks(batch_size = 100). Our measurements focused on the duration of basecalling alone, excluding the duration of subsequent necessary steps in adaptive sampling, such as barcode assignment (here, via alignment) and decision-making processes. Performance testing was conducted on a Windows-based user-grade system. Additionally, we performed a comparative analysis of the fast model configuration (case 1) on a Linux system to assess platform-specific performance characteristics. Due to technical limitations, we were unable to establish concurrent live basecalling with adaptive sampling on the Linux platform.

Hardware configuration
- CPU: AMD Ryzen 7 5800X3D
- RAM: 32GB
- GPU: NVIDIA RTX3070

Software configuration
- Operating System1: Windows 11 23H2
- Operating System2: Ubuntu Linux 22.04.3 LTS
- Dorado server: 7.6.8
- MinKNOW: 24.11.8
- minknow_api: 6.2.1
- readfish: 2024.3.0
- read_until_api: 3.4.1

The read_until package was additionally patched to force the use of read.id instead of read.number for compatibility with MinKNOW (see https://github.com/KleistLab/WarpDemuX).

Results
Our analysis revealed consistent performance patterns across the tested configurations (Supplementary Fig. 34 and Supplementary Table 19). In addition, for all configurations, measured latencies were independent of the number of reads per basecalling execution, which in practice constituted of 1–30 concurrently basecalled reads per execution. The latency of adaptive sampling basecalling with the fast model configuration (case 1, low accuracy) consistently was around 430 and 530 milliseconds, on Linux and Windows respectively. When utilizing the high accuracy configuration, (case 2) we observed an increased latency of 800 milliseconds, reflecting the additional computational over of the high accuracy basecalling model. The concurrent processing configuration (case 3) exhibited a total latency of 1000 milliseconds. In comparison, WarpDemuX's raw signal-based approach has a median latency of 41ms per read (time from start of analysis to rejection, mean 55ms, see Supplementary Note 6), including barcode assignment and decision-making with concurrent high accuracy live basecalling.

Discussion
Our analysis reveals fundamental performance differences between basecalling-based and signalbased approaches to adaptive sampling in nanopore sequencing. The observed order-of-magnitude disparity in processing latency—with basecalling-based methods requiring 400–1000 milliseconds compared to 41 milliseconds (median, see Supplementary Fig. 23) for

WarpDemuX' signal-based analysis—has significant implications for sequencing efficiency and throughput. This performance gap stems from the inherent latency of GPU-based basecalling, compared to the direct signal processing approach employed by WarpDemuX.

The latency differential becomes particularly critical for adaptive sampling in the context of direct RNA sequencing. Given the typically shorter length of RNA transcripts compared to DNA molecules, rapid decision-making is essential for enrichment. The extended processing time required by basecalling-based approaches means that target molecules occupy the pore for longer periods before rejection decisions can be implemented, thus decreasing the achievable enrichment and potentially limiting the practical utility of adaptive sampling for RNA applications. In the future, further optimization of basecalling approaches for real-time applications may address these challenges.

Beyond latency considerations, our findings highlight important practical constraints in sequencing workflow optimization. The substantial GPU computational demands of basecalling create a resource allocation challenge: users must either invest in additional GPU infrastructure or accept compromises in real-time sequencing by disabling live basecalling during adaptive sampling runs. This trade-off is explicitly acknowledged in Oxford Nanopore's technical documentation, which cautions about reduced enrichment efficiency when attempting concurrent basecalling and adaptive sampling operations (https://nanoporetech.com/document/ adaptive-sampling, accessed Jan 9th 2025). In contrast, WarpDemuX's signal-based approach operates efficiently without GPU requirements.

Supplementary Note 10. Number of training instances per barcode

During WarpDemuX training, we selected the number of training instances per barcode based on the average 10-fold cross-validated model performance on the validation set. The model reached optimal performance at a small number of training instances per barcode (Supplementary Fig. 32a), while run time kept increasing with the number of training instances as expected (Supplementary Fig. 32b). The model's performance was found to be robust with as few as 400 training instances per barcode.

Supplementary references

1. Smith, M. A. et al. Molecular barcoding of native RNAs using nanopore sequencing and deep learning. *Genome Research* **30**, 1345–1353 (2020).
2. Chang, C.-C. & Lin, C.-J. LIBSVM: A library for support vector machines. *ACM Transactions on Intelligent Systems and Technology* **2**, 1–27 (2011).
3. Pedregosa, F., Varoquaux, G., Gramfort, A., Michel, V. & Thirion. Scikit-learn: Machine learning in Python. *Journal of Machine Learning Research* **12**, 2825–2830 (2011).
4. Meert, W. *et al*. DTAIDistance. Zenodo (2022). doi: https://doi.org/10.5281/zenodo.1202378
5. Somervuo, P. et al. BARCOSEL: a tool for selecting an optimal barcode set for high-throughput sequencing. *BMC Bioinformatics* **19**, 257 (2018).
6. Bohn, P., Gribling-Burrer, A.-S., Ambi, U. B. & Smyth, R. P. Nano-DMS-MaP allows isoform-specific RNA structure determination. *Nat Methods* **20**, 849–859 (2023).
7. Krause, M. Flongle DirectRNA Library preparation https://dx.doi.org/10.17504/protocols.io.bcwcixaw.
8. Jalkanen, A. L., Coleman, S. J. & Wilusz, J. Determinants and implications of mRNA poly(A) tail size –Does this protein make my tail look big? *Seminars in Cell & Developmental Biology* **34**, 24–32 (2014).
9. Legnini, I., Alles, J., Karaiskos, N., Ayoub, S. & Rajewsky, N. FLAM-seq: full-length mRNA sequencing reveals principles of poly(A) tail length control. *Nature Methods* **16**, 879–886 (2019).
10. Krause, M. et al. tailfindr: alignment-free poly(A) length measurement for Oxford Nanopore RNA and DNA sequencing. *RNA* **25**, 1229–1241 (2019).
11. Begik, O. et al. Nano3P-seq: transcriptome-wide analysis of gene expression and tail dynamics using end capture nanopore cDNA sequencing. *Nature Methods* **20**, 75–85 (2023).
12. Liu-Wei, W. et al. Sequencing accuracy and systematic errors of nanopore direct RNA sequencing. *BMC Genomics* **25**, 528 (2024).
13. Payne, A. et al. Readfish enables targeted nanopore sequencing of gigabase-sized genomes. *Nature Biotechnology* **39**, 442–450 (2021).
